# A novel adaptor protein, ARAP, orchestrates Fcγ receptor signaling in mouse dendritic cells via Rap1 activation and NF‐κB‐mediated cytokine production

**DOI:** 10.1111/imcb.70133

**Published:** 2026-05-24

**Authors:** Chohee Park, Mi Jin Yu, Jong Ran Lee

**Affiliations:** ^1^ Department of Life Science, College of Natural Sciences Ewha Womans University Seoul Korea

**Keywords:** Adaptor protein, ARAP, dendritic cell, Fcγ receptor signaling

## Abstract

Fcγ receptors (FcγRs), key mediators of antibody‐dependent immune responses, are critical for dendritic cell (DC) activation and adaptive immunity initiation. Although the general importance of FcγR signaling is well established, the specific intracellular pathways remain insufficiently defined. ARAP (activation‐dependent, raft‐recruited ADAP‐like phosphoprotein) is a novel adaptor protein identified in T‐cell receptor signaling pathways. ARAP is expressed across various tissues and cell types, including DCs. To address the functional significance of ARAP, homologous recombination was performed to generate mice with an *ARAP* deficiency. In this study, we investigated the role of ARAP in FcγR‐mediated DC function using *ARAP*‐deficient mice. The results revealed that bone marrow‐derived DCs from *ARAP*‐deficient mice exhibited defects in FcγR‐mediated proximal signaling and integrin‐mediated adhesion. In addition, *ARAP*‐deficient DCs exhibited reduced nuclear translocation of NF‐κB p65 and decreased production of inflammatory cytokines. These findings establish ARAP as a selective and critical regulator of FcγR signaling, suggesting FcγR involvement in dendritic cell Rap1‐mediated adhesion and NF‐κB‐dependent cytokine responses.

## Graphical abstract

ARAP selectively regulates FcγR‐mediated signaling in dendritic cells. Loss of ARAP impairs phosphorylation of key signaling proteins, Rap1 activation, and integrin‐mediated adhesion, as well as NF‐κB p65 nuclear translocation and downstream cytokine production. In contrast, Rac1 activation and actin‐dependent processes, including actin polymerization and immune complex uptake, remain unaffected.

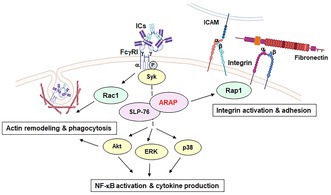

## INTRODUCTION

Dendritic cells (DCs), important immune cells of the hematopoietic system, are the most potent of the professional antigen‐presenting cells and play key roles in the initiation and regulation of adaptive immune responses.^1^, ^2^ Through antigen uptake, processing, and presentation, DCs act as a crucial bridge between the innate and adaptive immune systems.^3^, ^4^ Their activation is governed by a variety of pattern‐recognition and immunoregulatory receptors, among which Fcγ receptors (FcγRs) are key mediators of antibody‐dependent immune function.^5^, ^6^

The FcγRs are a family of cell surface receptors that bind to the Fc portion of monomeric and immune‐complexed IgG antibodies.^6^, ^7^ It is important to note that FcγR systems exhibit significant species‐specific differences in receptor composition, IgG subclass affinity, and cellular distribution.^8^, ^9^, ^10^ In mice, activating FcγRs include FcγRI, FcγRIII, and FcγRIV, which display distinct affinities for IgG subclasses such as IgG2a. In contrast, human FcγRs comprise FcγRI, FcγRIIA, FcγRIIB, and FcγRIIIA, with differing ligand specificities and cellular distribution. The present study focuses on murine FcγR signaling in DCs, and interpretations are made within this species‐specific context. Mouse FcγRI is a high‐affinity binding receptor for monomeric/immune‐complexed IgG.^6^, ^11^, ^12^ IgG2a efficiently engages activating murine FcγRs, particularly FcγRI and FcγRIV, thereby providing a robust model for FcγR‐mediated signaling in murine DCs.^11^, ^12^, ^13^

The activation of FcγR signaling occurs via immunoreceptor tyrosine‐based activation motifs (ITAMs), located in the cytoplasmic domain of the associated common γ‐chain.^14^, ^15^, ^16^ These ITAMs are phosphorylated upon receptor cross‐linking, leading to the recruitment of spleen tyrosine kinase (Syk) and the initiation of downstream signaling cascades, including the MAPK pathway, PI3K/Akt signaling, and regulation of small GTPases, such as Rac1 and Rap1, as well as transcription factors such as NF‐κB.^14^, ^15^, ^16^ Ultimately, these signals control the maturation and immunogenicity of the DCs by regulating antibody‐dependent cellular cytotoxicity and phagocytosis, antigen presentation, and the release of chemokines and cytokines.^1^, ^2^, ^3^, ^4^ Despite the importance of these pathways, and therefore these receptors, the full repertoire of intracellular regulators that modulate FcγR signaling in DCs remains undescribed.

In this study, therefore, we investigated the role of a novel adaptor protein, ARAP (activation‐dependent, raft‐recruited ADAP‐like phosphoprotein; not to be confused with ARAP1), in FcγR‐mediated signaling pathways, specifically that of the high‐affinity activating receptor, FcγRI. In a previous study,^17^ ARAP was identified in T cells as an adaptor protein with sequence similarity to ADAP (adhesion‐ and degranulation‐promoting adaptor protein), a key regulator of integrin‐mediated adhesion and immune synapse formation.^18^, ^19^ ARAP was shown to be required for proximal T‐cell receptor (TCR) signaling and for inside‐out signaling pathways that control integrin activation and T‐cell adhesion.^17^, ^20^ Importantly, these signaling modules, linking receptor engagement to cytoskeletal rearrangement and adhesion, are also central to FcγR signaling in myeloid cells.^14^, ^15^, ^16^ Furthermore, ARAP is broadly expressed across multiple immune cell types,^17^ suggesting that its function may not be restricted to T cells. Based on these observations, we hypothesized that ARAP may play a conserved role in FcγR‐mediated signaling, and therefore investigated its function in DCs using ARAP‐deficient mice. We used bone marrow‐derived dendritic cells (BMDCs) from *ARAP*‐knockout (KO) mice to investigate the potential changes in Rap1 activation, NF‐κB nuclear translocation, cytokine production, and integrin‐mediated adhesion.

Identifying novel components of FcγR signaling pathways in DCs is essential for a better understanding of how antibody‐mediated immune responses are regulated at the molecular level. The therapeutic manipulation of FcγRs through monoclonal antibodies or Fc‐engineered biologics will require a detailed understanding of how these receptors influence antigen‐presenting cell behavior. We propose that the adaptor protein ARAP is critical for FcγR‐mediated inside‐out signaling, resulting in DC adhesion and NF‐κB‐dependent cytokine production in DCs. These findings expand the current understanding of FcγR signaling in DCs and provide new insight into potential targets for immune modulation.

## RESULTS

### ARAP was involved in FcγR‐induced proximal signaling

**Figure 1:**
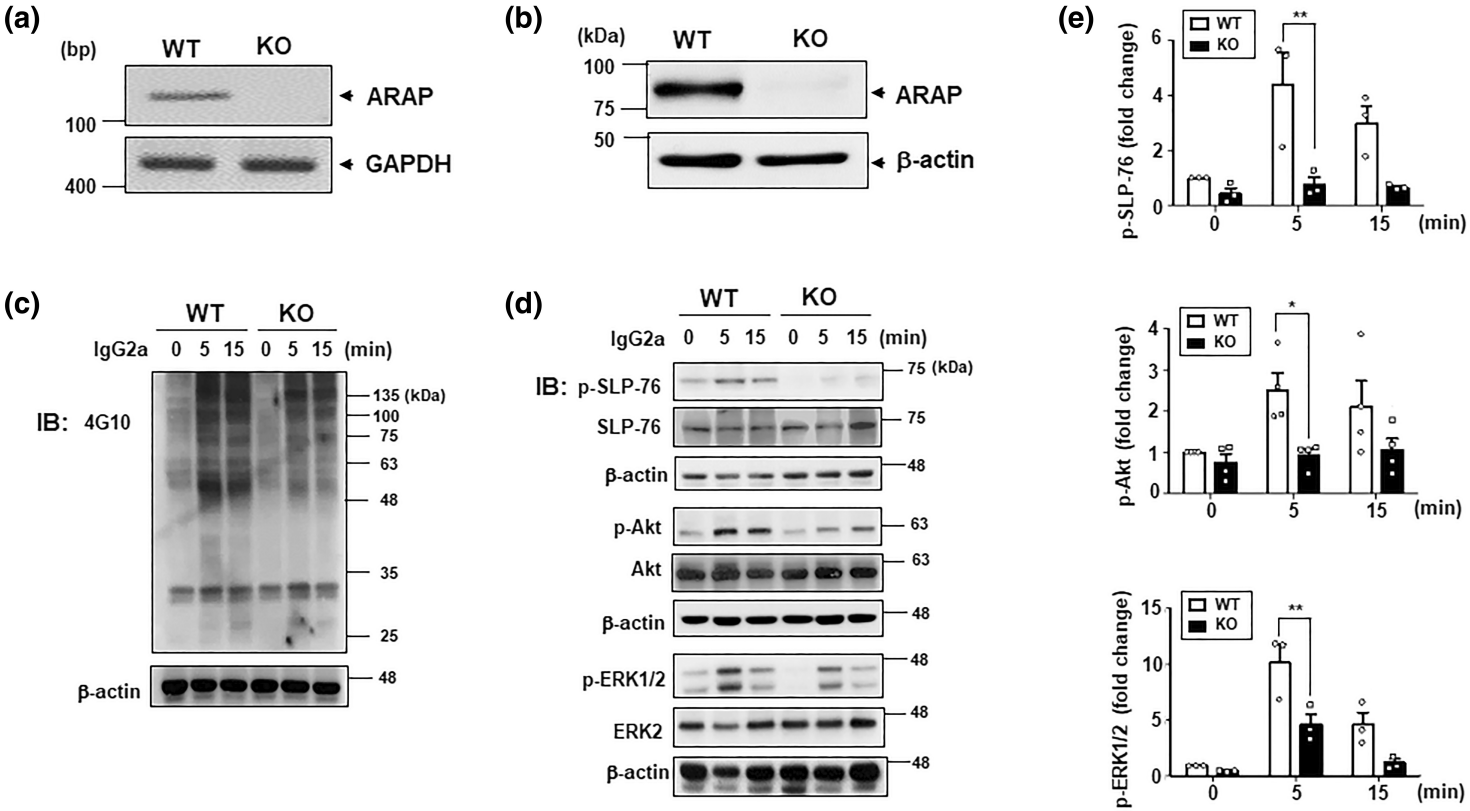
ARAP is expressed in BMDCs and contributes to FcγR‐induced proximal signaling. **(a)** RT‐PCR analysis of *ARAP* gene expression in WT and KO BMDCs. GAPDH was used as an internal control. **(b)** Immunoblotting (IB) analysis confirming the absence of ARAP expression in KO BMDCs. β‐Actin was used as a loading control. Data are representative of three independent experiments. ARAP, activation‐dependent, raft‐recruited ADAP‐like phosphoprotein. **(c)** Immunoblot analysis of global tyrosine phosphorylation following FcγR cross‐linking (IgG2a) in WT and KO BMDCs at the indicated time points. Overall tyrosine phosphorylation was reduced, but not abolished, in ARAP‐KO BMDCs compared to WT cells. β‐actin was used as a loading control. **(d)** Immunoblot analysis showing reduced phosphorylation levels of SLP‐76, Akt, and ERK1/2 in ARAP‐KO BMDCs compared to WT BMDCs. Corresponding total protein levels (SLP‐76, Akt, ERK1/2) are shown for each blot. β‐actin was included as a loading control. Data are representative of three independent experiments. **(e)** Quantitative analysis of phosphorylation of SLP‐76 (p‐SLP‐76), Akt (p‐Akt), and ERK1/2 (p‐ERK1/2). Band intensities were measured using ImageJ, and phosphorylation levels were normalized to their respective total protein levels. Values were expressed relative to unstimulated WT BMDCs. Data represent the mean ± s.e.m. of three independent experiments. Statistical significance was determined by two‐way ANOVA and Bonferroni's *post hoc* tests. *P < 0.05, **P < 0.01.

To establish the relevance of ARAP in DCs and to validate the genetic model used in this study, we first examined its expression in BMDCs derived from wild‐type (WT) and KO mice. We performed RT‐PCR and Western blotting using total BMDC lysates from WT and KO mice. RT‐PCR confirmed the absence of *ARAP* mRNA in KO BMDCs, whereas robust expression was detected in the WT cells (Figure 1a, Supplementary figure 1a). Similarly, Western blotting demonstrated complete loss of ARAP protein expression in the KO BMDCs (Figure 1b, Supplementary figure 1b).

Having confirmed the expression of ARAP in WT BMDCs and its complete absence in KO cell, we next investigated whether loss of ARAP affects FcγR‐medicated signaling. We assessed FcγR signaling competence by stimulating the BMDCs via the cross‐linking of FcγRs with the IgG2a + antimouse IgG complex. Total tyrosine phosphorylation patterns were analyzed by immunoblotting with the antiphosphotyrosine antibody (4G10), which detects multiple tyrosine‐phosphorylated proteins (Figure 1c, Supplementary figure 1c). For semiquantitative analysis, signal intensities were measured within defined molecular weight ranges (>100 kDa, 100–40 kDa, and <40 kDa) using ImageJ and normalized to β‐actin as a loading control (Supplementary figure 1c). Among these, a significant reduction in signal intensity was observed in the 100–40 kDa range in ARAP‐deficient cells compared to WT controls (Supplementary figure 1c). This molecular weight range includes several key signaling molecules downstream of FcγR, such as SLP‐76, Akt, and ERK1/2. Consistent with the 4G10 analysis showing reduced tyrosine phosphorylation within the 40–100 kDa range at both 5 and 15 min (Supplementary figure 1c), phosphorylation of these proteins was further assessed individually. Phosphorylation of SLP‐76, Akt, and ERK1/2 was significantly reduced at 5 min in ARAP‐deficient cells, whereas the differences were less pronounced at 15 min potentially due to the transient nature of their activation (Figure 1d, e; Supplementary figure 1d). In contrast, the sustained reduction observed in the 4G10 signal at 15 min may reflect contributions from additional tyrosine‐phosphorylated proteins within the same molecular weight range.

### ARAP loss selectively impairs Rap1 activity but not Rac1 activity

**Figure 2:**
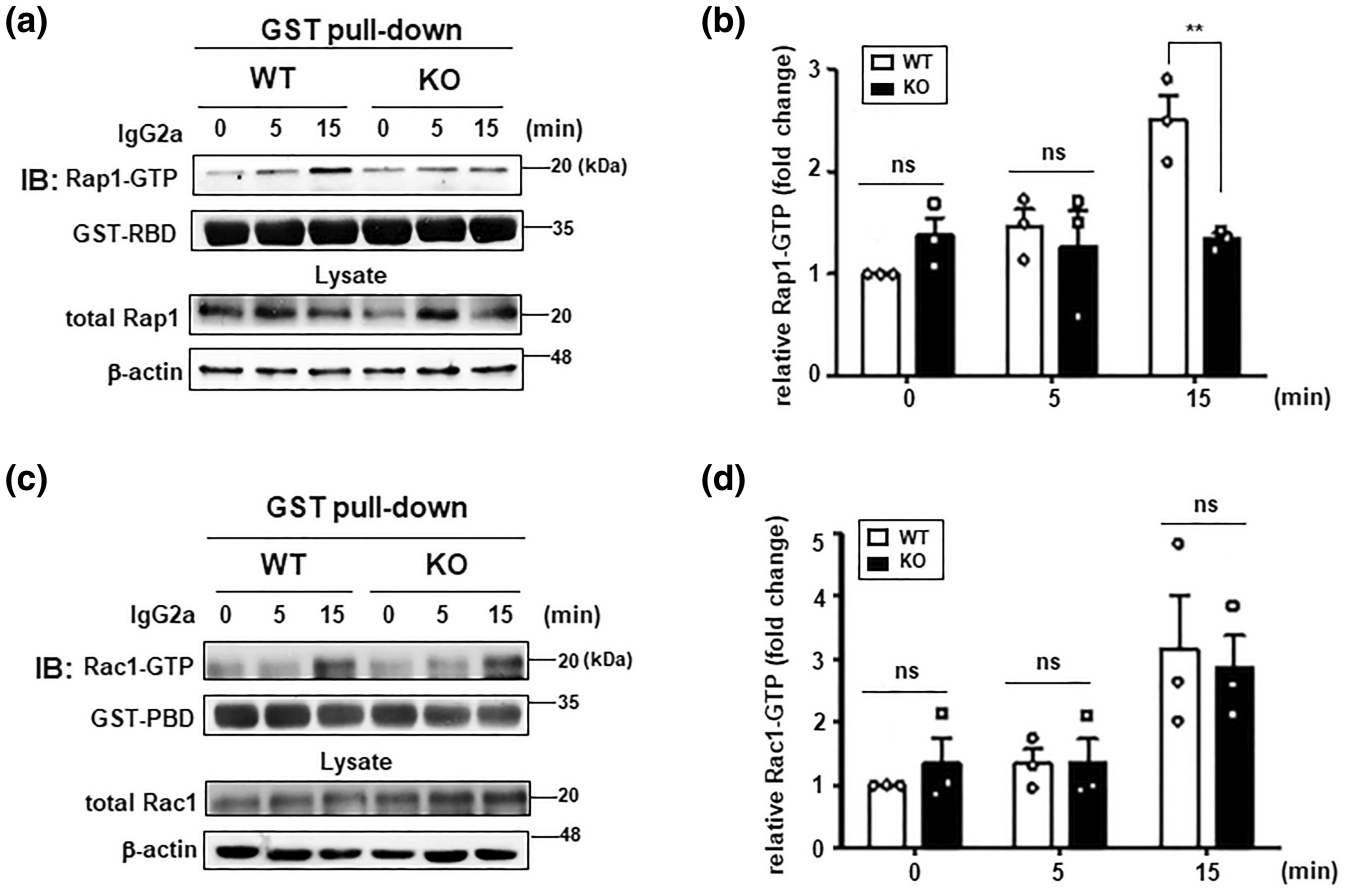
Differential regulation of Rap1 and Rac1 activity by ARAP. WT and ARAP‐KO BMDCs were FcγR cross‐linked at 37°C for the indicated durations. Cells were lysed and the lysates were incubated with GST‐fusion protein beads at 4°C for 20 min. The proteins that were pulled down by the beads were resolved by SDS‐PAGE, followed by IB. IB also showed equal amounts of fusion proteins used with anti‐GST antibody. The total amounts of each GTPase and β‐actin in the lysates are also shown. **(a, b)** GTP‐bound Rap1 (Rap1‐GTP) was detected by GST‐RalGDS‐RBD pull‐down assay. Quantification was performed based on the intensity of Rap1‐GTP bands and normalized to β‐Actin levels from the corresponding whole‐cell lysates, which served as loading controls. **(c, d)** GTP‐bound Rac1 (Rac1‐GTP) was detected by GST‐PAK‐PBD pull‐down assay. Band intensities of Rac1‐GTP were quantified and normalized to β‐actin levels in the corresponding whole‐cell lysates. IB results are representative of three independent experiments. Densitometric quantification is shown as fold changes compared with the Rap1‐GTP **(b)** or Rac1‐GTP **(d)** levels of unstimulated WT BMDCs. Data represent the mean ± s.e.m. of three independent experiments. Statistical significance was determined by two‐way ANOVA and Bonferroni's *post hoc* tests. **P < 0.01; ns, not significant.

To investigate whether impaired signaling in KO BMDCs is associated with altered small GTPase activity, we measured the activation of Rap1 and Rac1 following FcγR engagement. Active Rap1 (GTP‐bound form) expression was substantially reduced in KO BMDCs, as measured by pull‐down assays using a Rap1‐binding domain (RBD) probe (Figure 2a, Supplementary figure 2a). Quantitative analysis revealed fold changes in Rap1 activity after FcγR stimulation in WT BMDCs, but not in KO BMDCs (Figure 2b). To further address the kinetics of Rap1 activation, we extended the time course to 30 min. Rap1 activation peaked at 15 min in WT cells and declined thereafter, indicating transient activation kinetics (Supplementary figure 2c, d). Therefore, the reduced Rap1 activation in ARAP‐deficient cells was most evident at early time points, suggesting that ARAP contributes to the efficient initiation of Rap1 activation rather than its sustained signaling.

In contrast, Rac1 activity, as assessed using a PAK1‐binding domain (PBD) pull‐down assay, was not considerably different between the WT and KO BMDCs (Figure 2c, Supplementary figure 2b). Quantitative analysis did not reveal changes in Rac1 activity after FcγR cross‐linking between WT and KO BMDCs (Figure 2d). These results indicate that ARAP specifically regulates the Rap1 pathway, without broadly affecting GTPase activation.

### Actin polymerization and antigen uptake remained intact in the absence of ARAP

**Figure 3:**
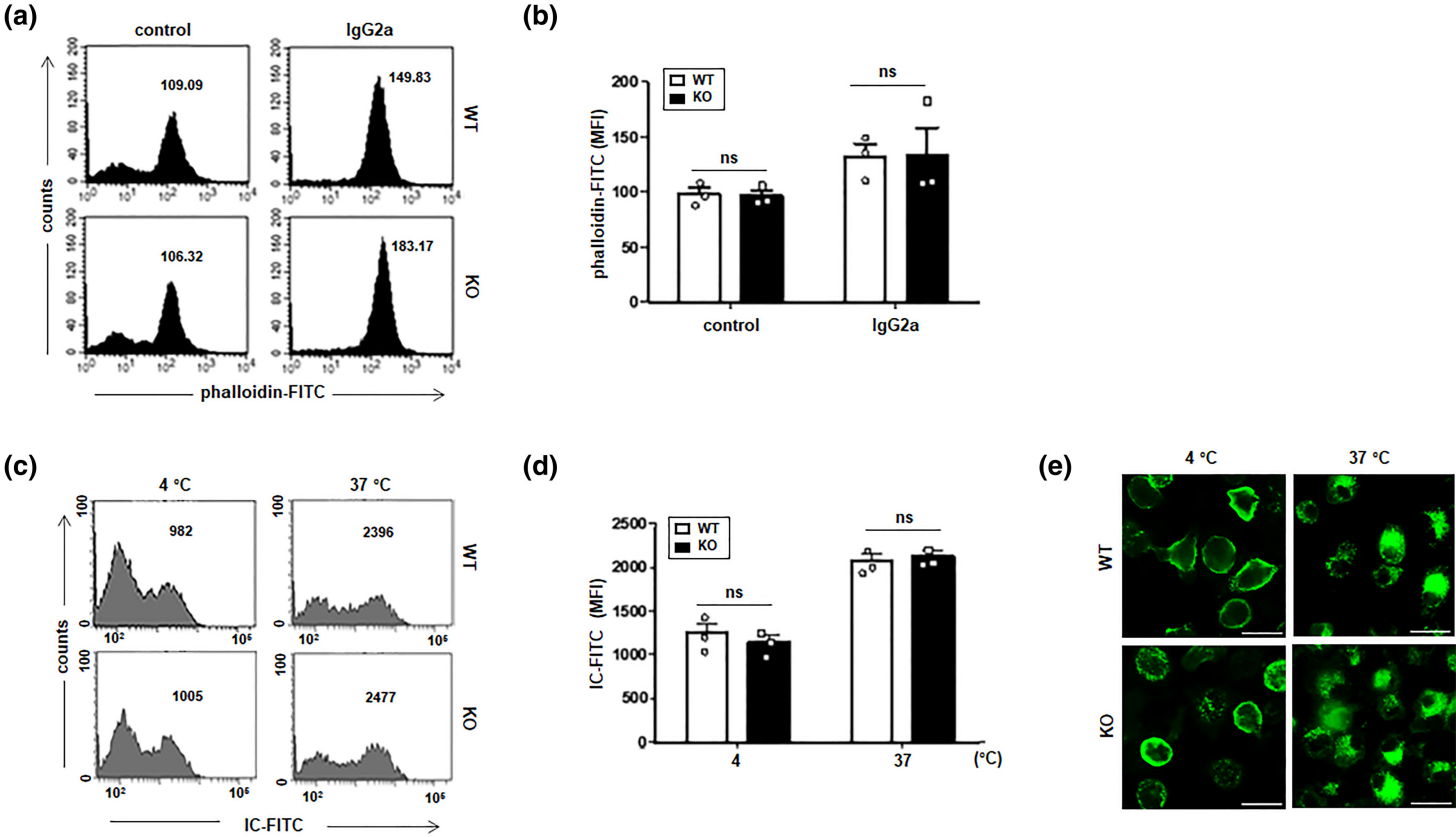
Actin polymerization and IC uptake were preserved in ARAP‐KO BMDCs. **(a, b)** Flow cytometric analysis of F‐actin levels following FcγR stimulation. **(a)** WT and ARAP‐KO BMDCs were cultured with plate‐bound IgG2a for 24 h, stained with FITC‐conjugated phalloidin, and analyzed for cellular polymerized F‐actin by flow cytometry. The numbers indicate the MFIs of the cells in the unstimulated control and IgG2a‐stimulated samples. Histograms are representative of three separate experiments. **(b)** The MFIs are presented as the mean ± s.e.m. of three separate experiments. Significant comparisons were determined by two‐way ANOVA (ns, no significance). **(c–e)** Quantification of IC uptake at 37°C. WT and ARAP‐KO BMDCs were incubated with fluorescent ICs at 4°C or 37°C for 30 min and fixed with 4% PFA. **(c)** IC uptake was analyzed by flow cytometry. The numbers indicate the MFIs of cells incubated at 4°C or 37°C. The histograms are representative of three separate experiments. **(d)** The MFIs are presented as the mean ± s.e.m. of three separate experiments. Significant comparisons were determined using two‐way ANOVA (ns, no significance). **(e)** The cells incubated with fluorescent ICs at 4°C or 37°C were analyzed using fluorescence microscopy. Representative images are shown. Scale bars = 10 μm.

Given the preserved Rac1 activity in KO BMDCs, we next investigated cytoskeletal dynamics by assessing actin polymerization following FcγR stimulation. BMDCs were stained with fluorescent phalloidin after FcγR cross‐linking, and the fluorescence intensity of F‐actin was measured via flow cytometry. Both WT and KO BMDCs showed comparable increase in F‐actin formation, with no significant differences in mean fluorescence intensity (MFI) between the genotypes (Figure 3a, b, Supplementary figure 3a).

We further examined FcγR‐mediated antigen uptake using IgG2a‐cross‐linked fluorescent immune complexes (ICs). WT and KO BMDCs internalized the ICs at 37°C with similar efficiency, as quantified by flow cytometry (Figure 3c, d, Supplementary figure 3b). The MFI of the cells after IC internalization was not notably different between the WT and KO BMDCs, indicating that endocytic responses were preserved in KO BMDCs. A similar efficiency of IC internalization was also shown by the immunofluorescence detection of WT and KO BMDCs after incubation with the fluorescent ICs (Figure 3e, Supplementary figure 3c). The ICs were surface‐bound at 4°C and were internalized in the interiors of the WT and KO BMDCs at 37°C. These results suggest that Rac1‐dependent actin remodeling and antigen uptake remain functional in the absence of ARAP.

### FcγR‐mediated adhesion is impaired in knockout BMDCs

**Figure 4:**
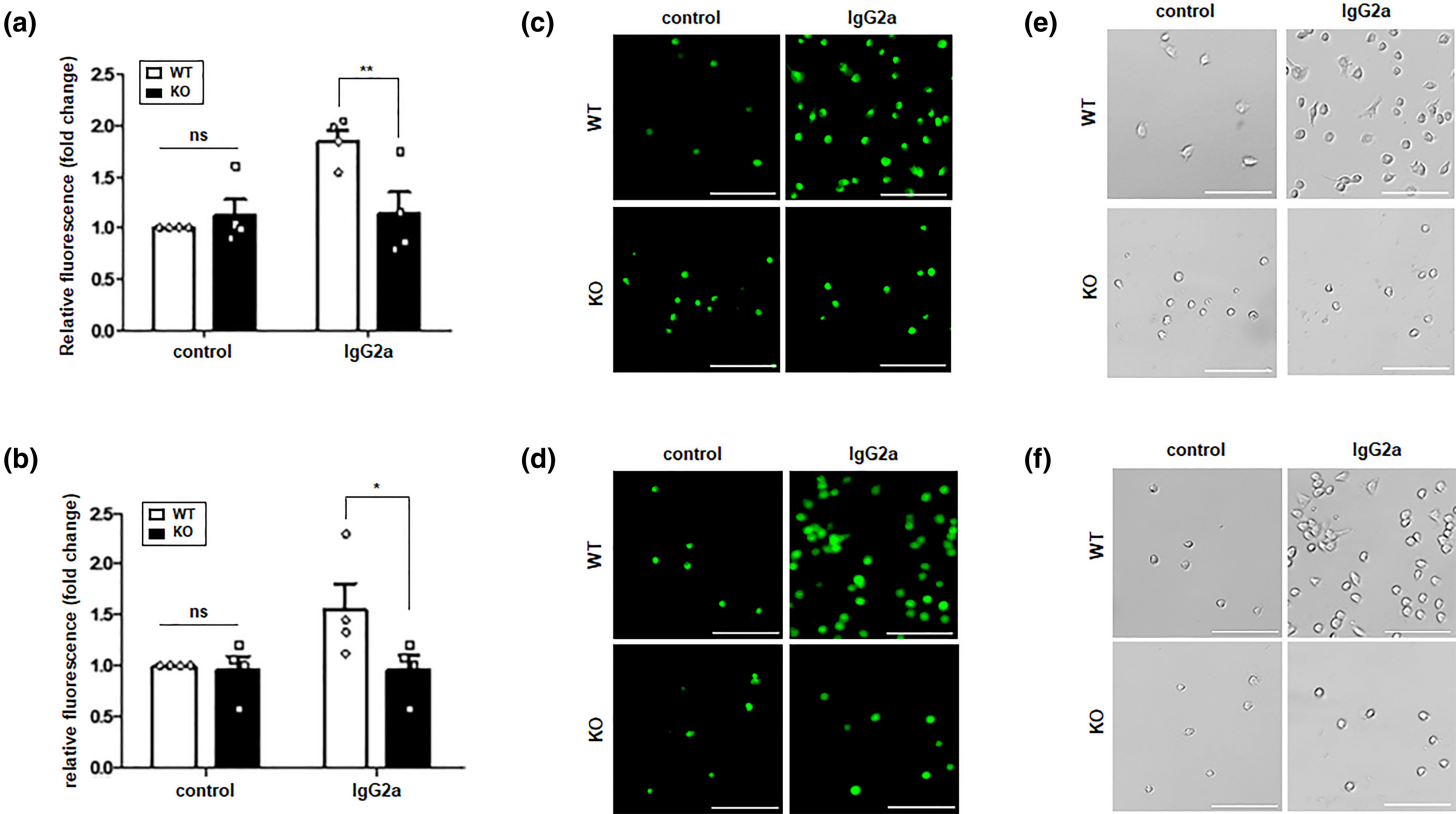
Adhesion defects in ARAP‐KO BMDCs. Calcein‐labeled WT and ARAP‐KO BMDCs were either left unstimulated (control) or FcγR‐stimulated (IgG2a) at 37°C for 30 min. Cells were seeded into the wells of a plate coated with fibronectin **(a, c, e)** or ICAM‐1‐Fc **(b, d, f)**. **(a, b)** Fluorescence intensity correlates with the number of adherent calcein‐labeled cells and is presented as relative values normalized to unstimulated WT BMDCs. Each individual dot represents the mean of technical triplicates from one independent experiment, and the data shown are compiled from multiple independent experiments. The data are presented as the mean ± s.e.m. Statistical significance was analyzed by two‐way ANOVA with Bonferroni's *post hoc* tests (*P < 0.05, **P < 0.01; ns, no significance). **(c, d)** The adherent cells after 2 h of incubation at 37°C were analyzed by microscopy. Representative fluorescence images are provided to qualitatively support the adhesion phenotype. Scale bars = 100 μm. **(e, f)** Bright‐field images of the same samples showing morphological changes after FcγR stimulation. Representative images are shown. Scale bars = 100 μm.

To determine whether impaired Rap1 activation affected integrin‐mediated adhesion, we assessed the ability of FcγR‐stimulated BMDCs to adhere to fibronectin‐ or ICAM‐1‐coated surfaces. In WT cells, FcγR cross‐linking considerably increased the adhesion to fibronectin, as measured by calcein staining (Figure 4a). In contrast, KO BMDCs exhibited markedly reduced adhesion under the same conditions (Figure 4a). Similarly, increased adhesion to ICAM‐1 was observed in FcγR‐stimulated WT cells compared with KO BMDCs (Figure 4b).

Microscopic examination of the bound cells in fibronectin‐ or ICAM‐1‐coated wells showed that KO cells adhered poorly and exhibited less cell spreading and fewer uropods than WT control cells when FcγR was cross‐linked (Figure 4c–f, Supplementary figure 5a–d). These data indicate that ARAP positively regulates the magnitude of FcγR‐induced adhesion, likely through regulation of the Rap‐1‐integrin axis. Notably, these defects occurred in the absence of changes in actin polymerization (Figure 3a, b, Supplementary figure 3a) or integrin surface expression (Supplementary figure 6). In addition, basal adhesion was similar between WT and KO cells in the absence of stimulation, whereas a significant difference was observed only following FcγR activation. Thus, the reduced adhesion observed in ARAP‐KO BMDCs is not due to altered expression of adhesion molecules, but rather suggests a defect in inside‐out signaling pathways regulating adhesion.

### Antigen uptake is preserved but T‐cell activation is impaired in ARAP‐deficient DCs

**Figure 5:**
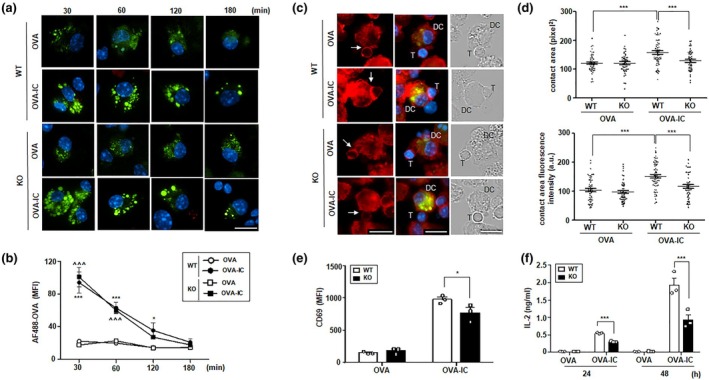
ARAP regulates T‐cell activation without affecting antigen uptake or processing in DCs. **(a)** Uptake and intracellular trafficking of soluble OVA and OVA immune complexes (OVA‐ICs) in WT and ARAP‐KO DCs. Alexa Fluor 488‐conjugated OVA (AF488‐OVA) was incubated with anti‐OVA IgG2a to generate OVA‐ICs. Cells were pulsed with AF488‐OVA or AF488‐OVA‐ICs at 37°C for 30 min, washed, and chased for the indicated times (up to 3 h). Cells were analyzed by fluorescence microscopy. Representative images show comparable uptake and intracellular distribution of OVA‐ICs in WT and ARAP‐KO DCs. Scale bar = 25 μm. **(b)** Quantification of OVA‐IC uptake. Mean fluorescence intensity (MFI) of intracellular OVA was quantified using ImageJ. WT and KO cells pulsed with OVA or OVA‐ICs were randomly selected (30 cells per condition) from three independent experiments. Data are presented as the mean ± s.e.m. Statistical significance was determined using one‐way ANOVA and Tukey's *post hoc* test. No significant differences were observed between WT and KO DCs. Significant comparisons are noted as ^^^*P* < 0.001 (KO; OVA *versus* OVA‐IC), ***P < 0.001 (WT; OVA *versus* OVA‐IC), *P < 0.05 (WT; OVA *versus* OVA‐IC). **(c)** Analysis of DC‐T cell contact. WT or ARAP‐KO DCs pulsed with OVA or OVA‐ICs were cocultured with OT‐II T cells, and contact areas (arrows) were visualized by Actin accumulation at the DC‐T cell interface after 30 min. Conjugated T cells (T) were indicated by distinct size differences from DCs. Representative images show enhanced contact formation in WT DCs pulsed with OVA‐ICs. Red and blue indicate actin and DNA staining, respectively. Scale bar = 25 μm. **(d)** Quantification of DC‐T cell contact. Contact area was quantified using ImageJ by measuring the cell–cell interface (pixel^2^) or the fluorescence intensity (arbitrary units, a.u.) at the interface. Statistical significance was determined using one‐way ANOVA with Tukey's *post hoc* tests. Significant differences are indicated as ***P < 0.001. **(e)** T‐cell activation assessed by CD69 expression. OT‐II T cells were cocultured with OVA‐ or OVA‐IC‐pulsed DCs for 24 h and analyzed by flow cytometry. Reduced CD69 expression was observed in T cells stimulated with ARAP‐KO DCs pulsed with OVA‐ICs. Data are presented as mean ± s.e.m. from three independent experiments. Statistical significance was determined using two‐way ANOVA with Bonferroni's *post hoc* tests. Significant differences are indicated as *P < 0.05. **(f)** IL‐2 production by OT‐II T cells. Supernatants were collected at 24 and 48 h after coculture and analyzed by ELISA. ARAP‐KO DCs induced significantly lower IL‐2 production compared to WT DCs. Data are presented as mean ± s.e.m. from three independent experiments. Statistical significance was determined using two‐way ANOVA with Bonferroni's *post hoc* tests. Significant differences are indicated as ***P < 0.001.

To determine whether ARAP deficiency affects antigen uptake and processing, we first examined the internalization of soluble antigen and immune complexes (ICs) in DCs. Alexa Fluor 488‐conjugated ovalbumin (AF488‐OVA) was used either alone or in the form of ICs generated with anti‐OVA IgG2a. As expected, OVA‐ICs were efficiently internalized in both WT and ARAP‐deficient DCs, whereas uptake of soluble OVA was comparatively weak (Figure 5a, Supplementary figure 8). Importantly, no distinct differences were observed between WT and KO DCs in the extent or intracellular distribution patterns of OVA‐IC over time (Figure 5b), suggesting that ARAP is not required for FcγR‐mediated antigen uptake or intracellular trafficking.

We next assessed the functional consequence of ARAP deficiency on antigen presentation using OVA‐specific CD4^+^ T cells. WT or ARAP‐deficient DCs were pulsed with OVA or OVA‐ICs and cocultured with OT‐II T cells. We analyzed early DC‐T cell interaction. Notably, ARAP‐deficient DCs formed reduced contact areas with OT‐II T cells at 30 min after coculture, indicating impaired cell–cell interaction (Figure 5c, d, Supplementary figure 9). Consequently, ARAP‐deficient DCs induced significantly reduced T‐cell activation, as evidenced by decreased CD69 expression at 24 h and reduced IL‐2 production at both 24 and 48 h (Figure 5e, f, Supplementary figure 10).

Together, these results demonstrate that ARAP is dispensable for antigen uptake and likely antigen processing, including in the context of immune complexes, but is involved in modulating efficient T‐cell activation, potentially through the regulation of DC‐T cell interaction.

### ARAP loss impaired NF‐κB nuclear translocation and cytokine production in response to FcγR stimulation

**Figure 6:**
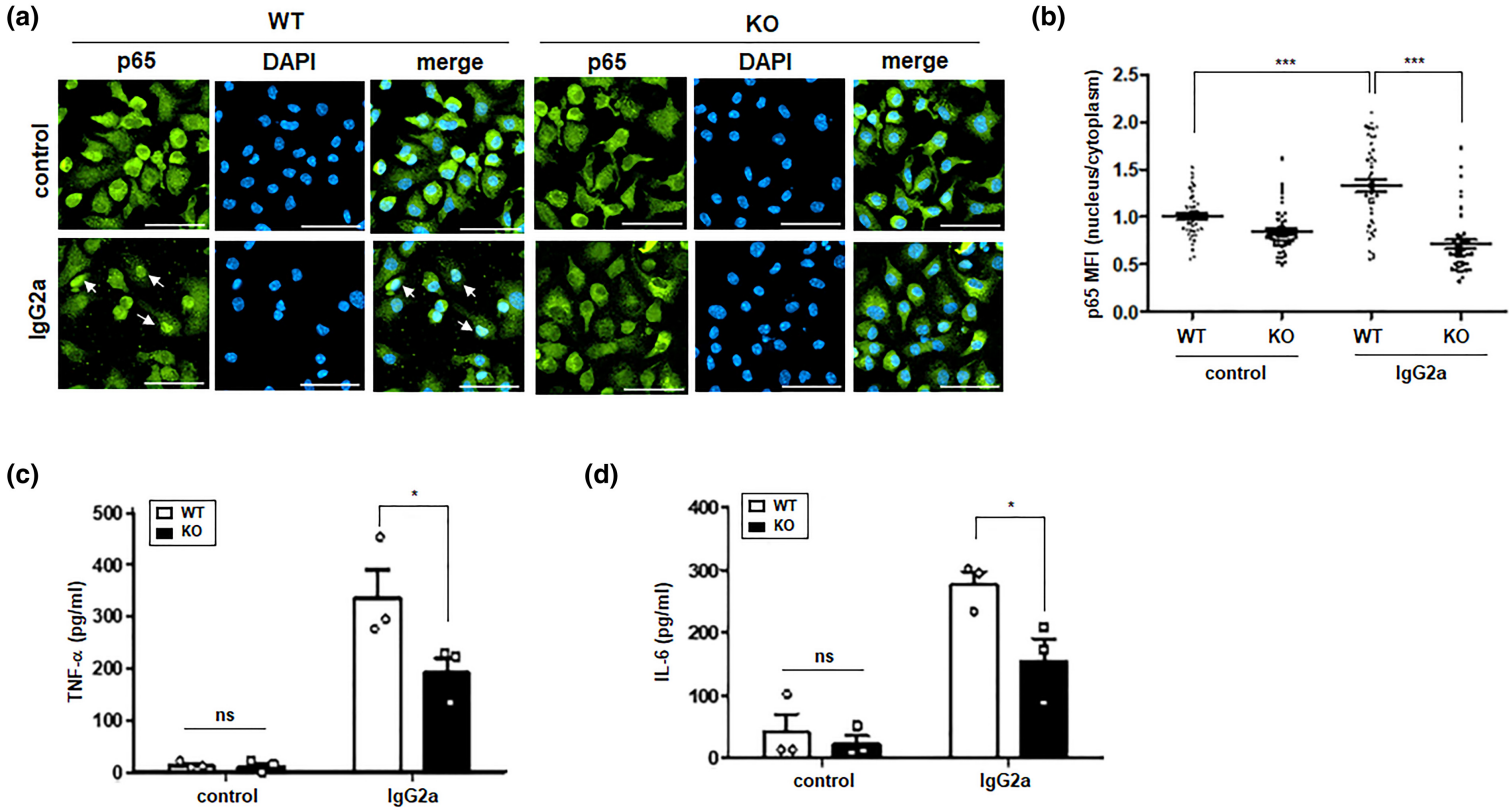
Impaired FcγR‐mediated NF‐κB nuclear translocation and cytokine production in ARAP‐KO BMDCs. **(a, b)** WT and KO BMDCs (2 × 10^5^) either alone or with IgG2a and antimouse IgG antibody were added to the coverslips and incubated at 37°C for 1 h. For an immunofluorescence assay of NF‐κB (p65), cells were fixed, permeabilized, and stained with anti‐p65 followed by FITC‐conjugated secondary antibodies. Cells were scored as p65‐positive nuclei only when p65 signal colocalized with DAPI staining. **(a)** Representative images showing FcγR‐stimulated NF‐κB nuclear translocation (arrows) in the BMDCs of WT and KO mice. The experiment was repeated twice. Scale bars = 50 μm. **(b)** MFI of the nucleus/cytoplasm of each cell. The data are the averaged intensities of 40 cells blindly selected from four random microscopic fields for each sample. The data are presented as the mean ± s.e.m. Statistical significance was determined by one‐way ANOVA with Tukey's *post hoc* tests. Significant comparisons are annotated as ***P < 0.001. **(c, d)** BMDCs from WT and KO mice were cultured in a plate with either media alone (control) or coated with IgG2a at 37°C for 24 h. The levels of TNF‐α **(c)** and IL‐6 **(d)** in the supernatants were compared using ELISA. The data represent the mean ± s.e.m. of three independent experiments. Statistical significance was analyzed using two‐way ANOVA with Bonferroni's *post hoc* tests. Significant comparisons are noted as *P < 0.05; ns, no significance.

To determine whether the impaired signaling observed in the KO BMDCs also extended to transcriptional responses, we assessed nuclear translocation of NF‐κB subunit p65 following FcγR cross‐linking. In the WT BMDCs, IgG2a stimulation induced rapid p65 accumulation in the nucleus, as detected by immunofluorescence (Figure 6a, b, Supplementary figure 11). In contrast, the KO BMDCs showed substantially reduced p65 nuclear translocation under the same condition (Figure 6a, b, Supplementary figure 11).

In‐line with this, ELISA of the culture supernatants revealed that TNF‐α and IL‐6 secretion in response to FcγR cross‐linking was significantly diminished in the KO BMDCs compared to the WT (Figure 6c, d). These results suggest that ARAP is not strictly required but rather contributes to the efficient activation of FcγR‐mediated signaling, adhesion, transcriptional activation and cytokine production.

## DISCUSSION

FcγRs play central roles in innate and adaptive immunity by their linking recognition of antibody‐opsonized targets to cellular activation programs. In DCs, FcγR‐mediated signaling promotes antigen uptake, cytokine production, cell migration, and integrin‐dependent adhesion, all of which are critical for efficient antigen presentation and T‐cell priming.^3^, ^4^, ^12^, ^13^, ^14^, ^15^ Although the general downstream signaling cascades of FcγRs have been studied in myeloid cells, the molecular adaptors that selectively modulate specific functional outputs, such as integrin activation versus actin remodeling, remain incompletely defined.

In this study, we identified a novel adaptor protein that is involved in modulating FcγR‐induced Rap1 activation and adhesion in DCs, while being dispensable for Rac1 activation, actin polymerization, and antigen uptake. Using BMDCs derived from KO mice lacking the ARAP adaptor protein, we found that IgG2a‐mediated FcγR stimulation led to markedly reduced phosphorylation of key signaling proteins, including SLP‐76, ERK, and Akt (Figure 1), which suggested that ARAP is a positive regulator of FcγR‐proximal signaling. Importantly, although Rac1 activity and downstream actin‐based functions such as polymerization and IC uptake remained intact, Rap1 activation was selectively impaired in KO BMDCs (Figures 2 and 3). This was accompanied by a significant reduction in adhesion to fibronectin‐ and ICAM‐1‐coated surfaces following FcγR cross‐linking, consistent with defective Rap1‐mediated integrin activation (Figure 4). The reduced Rap1 activation in ARAP‐deficient cells was most evident at early time points, suggesting that ARAP contributes to the efficient initiation of Rap1 activation rather than its sustained signaling. Additionally, the normal surface expression of integrins, together with the selective defect in stimulated adhesion, supports a role for ARAP in inside‐out signaling rather than in the regulation of adhesion molecule expression. Additional surface molecules may contribute to adhesion regulation; however, our data support a primary role for ARAP in signaling‐mediated modulation of integrin activation.

Although BMDCs provide a useful model, they do not fully reflect the heterogeneity of in vivo DC subsets. Our findings in splenic CD11c^+^ DCs partially validate the observed adhesion phenotype, supporting the physiological relevance of ARAP function (Supplementary figures 4 & 7).

Moreover, the dissociation between preserved antigen uptake and impaired T‐cell activation in ARAP‐deficient DCs suggests that ARAP primarily regulates downstream events such as DC‐T‐cell interaction rather than antigen processing itself (Figure 5). The adhesion defect observed in ARAP‐deficient DCs likely reflects impaired integrin activation and translates into reduced DC‐T‐cell interaction, ultimately leading to suboptimal T‐cell activation. Our study focused on antigens directly associated with immune complexes, and future studies will be required to determine whether ARAP also influences the processing or presentation of bystander antigens codelivered during FcγR engagement.

**Figure 7:**
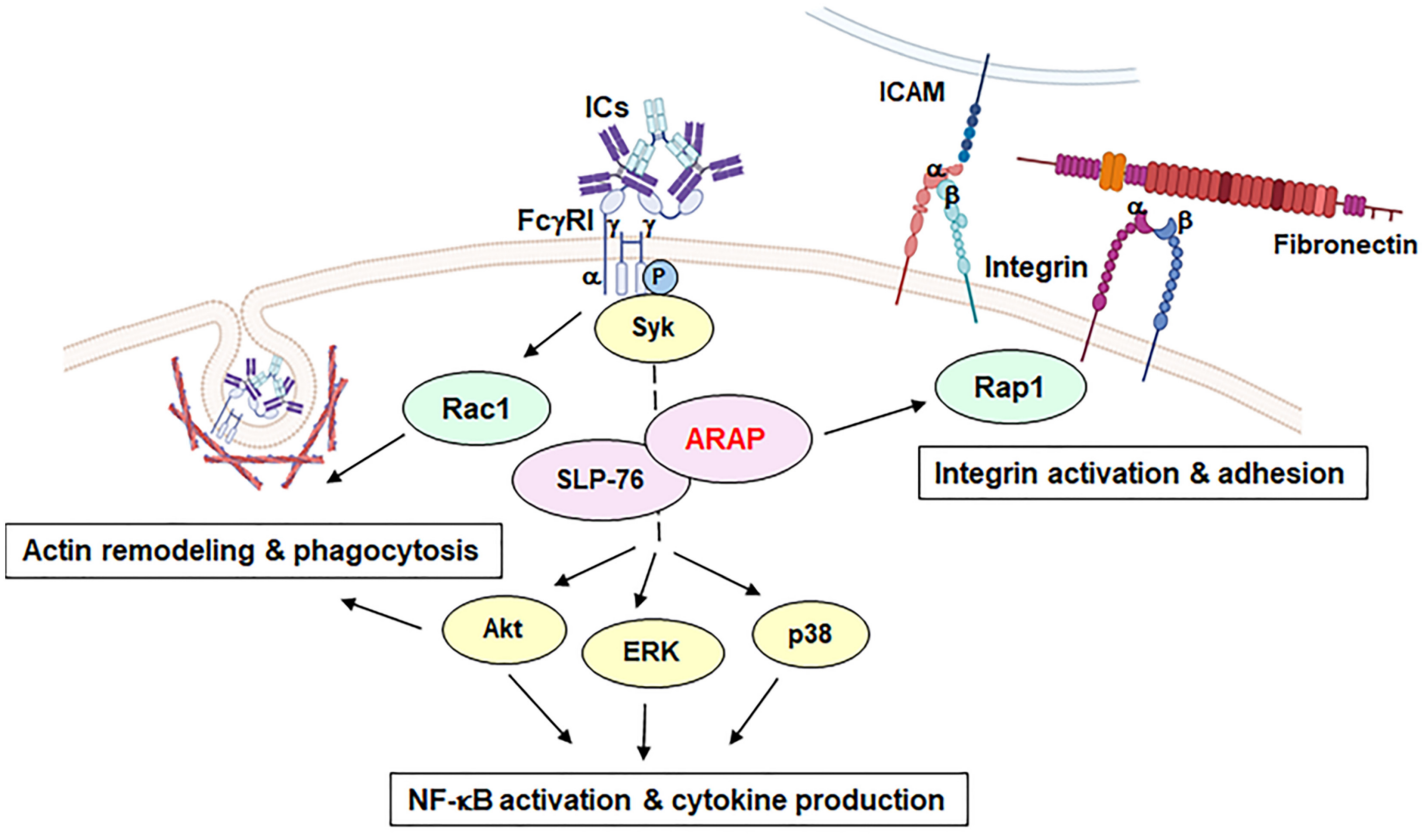
Model of the role of the ARAP adaptor protein in FcγR signaling: Rap1‐Mediated integrin adhesion and NF‐κB‐mediated cytokine production. In the absence of ARAP, signaling through FcγR, including phosphorylation of key signaling proteins (SLP‐76, Akt, p38, and ERK), Rap1 activation followed by integrin‐mediated adhesion, as well as NF‐κB p65 nuclear translocation followed by cytokine production, was inhibited. In contrast, Rac1 activity and downstream actin‐related functions, such as polymerization and IC uptake, remained intact.

These findings support a model in which ARAP functions as a specialized adaptor that links FcγR engagement to Rap1 activation and adhesion, but not to actin remodeling or uptake (Figure 7). This selective defect revealed a previously unappreciated degree of modularity in FcγR downstream signaling, in which distinct adaptor modules may independently govern discrete cellular outputs.

Consistent with its signaling role, the loss of ARAP also resulted in reduced nuclear translocation of the NF‐κB subunit, p65, and diminished production of TNF‐α and IL‐6 following FcγR engagement (Figure 6). This further supports the hypothesis that the ARAP adaptor protein integrates the signals necessary for proinflammatory cytokine responses (Figure 7). Interestingly, functions such as actin‐driven phagocytosis and IC uptake were not affected, suggesting that the ARAP protein specifically governs the signaling arms rather than the cytoskeletal machinery of FcγR pathways.

Although ARAP has not been previously linked to ITAM‐associated signaling in myeloid cells, its functional impact on FcγR‐mediated signaling suggests that it may represent a novel adaptor operating in this pathway. Notably, a similar signaling defect was observed in T cells derived from the same ARAP‐KO mice, including impaired phosphorylation of MAPK and Akt in response to TCR stimulation.^20^ Our data raise the possibility that ARAP participates in shared signaling mechanisms downstream of ITAM‐associated receptors, although this will require further validation. Both the TCR and FcγR share downstream components such as kinases and phosphoinositide pathways,^12^, ^14^, ^21^, ^22^, ^23^ suggesting the potential role of ARAP in a shared signaling scaffold. This notion is supported by the results of previous studies that demonstrated that adaptor proteins such as LAT and SLP‐76 are employed by both the TCR and FcγR pathways, albeit in a cell‐type‐dependent manner. SLP‐76, for example, is essential for FcγR‐mediated calcium flux and reactive oxygen species generation in neutrophils, but is dispensable in macrophages.^24^, ^25^ Similarly, LAT associates with the FcγR chain in monocytic cells and becomes phosphorylated upon FcγR cross‐linking in monocytic cells, but its expression was found to be minimal in murine macrophages.^26^ These findings underscore the modular and context‐dependent use of adaptors in immune receptor signaling. Our data extends this paradigm by identifying an uncharacterized adaptor protein that contributes to FcγR‐mediated signaling in DCs and may participate in a broader, shared adaptor network across leukocyte lineages.

Our results also complement previous studies on ADAP, another adaptor protein known to function in both TCR and integrin signaling.^27^, ^28^, ^29^, ^30^, ^31^ In DCs, ADAP deficiency impairs migration, polarization, and T‐cell priming in response to LPS stimulation, largely via defective β2 integrin (LFA‐1) activation.^32^, ^33^, ^34^ However, those studies focused primarily on Toll‐like‐receptor‐driven activation or integrin engagement and did not explore FcγR signaling pathways. Our results provide the first evidence of an adaptor protein that selectively regulates Rap1 activity downstream of FcγR engagement in DCs without broadly altering actin‐dependent processes. This contrast highlights a functional divergence between ADAP and ARAP: while ADAP integrates multiple signaling pathways leading to cytoskeletal remodeling,^35^, ^36^ ARAP appears to act specifically within the FcγR‐Rap1‐integrin axis. In addition, the selective impairment of adhesion despite preserved actin polymerization implies that Rap1‐mediated integrin activation may occur independently of, or in parallel with, actin remodeling in the context of FcγR signaling.

Collectively, our findings provide new insights into the compartmentalization of FcγR signaling in DCs and reveal ARAP as a novel component of the FcγR‐Rap1 axis that governs integrin‐dependent adhesion. These results broaden the understanding of how innate immune receptors orchestrate discrete functional outputs through adaptor specificity and may inform future strategies targeting Fc receptor pathways in immunomodulation or vaccination.

A key consideration in interpreting these findings is the species‐specific nature of FcγR systems. While murine FcγRs differ from their human counterparts in receptor composition and IgG subclass specificity,^8^, ^9^, ^10^ the core signaling architecture downstream of FcγR engagement, including ITAM‐dependent activation of Syk, MAPK, PI3K‐Akt, and NF‐κB, is highly conserved across species.^23^ Therefore, although the present findings are derived from a murine model, the identification of this adaptor protein as a regulator of FcγR‐mediated signaling may reflect a conserved mechanism applicable to human immune cells. These considerations highlight the importance of extending future studies to human DCs to validate the translational relevance of this adaptor protein in FcγR‐mediated immune responses.

## METHODS

### Mice

The generation of ARAP‐KO mice and the DNA genotyping were performed as described previously.^20^ WT, ARAP‐KO, and OT‐II TCR transgenic mice were bred and maintained under specific pathogen–free conditions at the animal facility of Ewha Laboratory Animal Genomic Center in accordance with institutional guidelines. All mouse protocols were approved by the Ewha Institutional Animal Care and Use Committee. Mice aged 8–13 weeks were used in all experiments.

### Antibodies and reagents

Mouse IgG2a and goat antimouse IgG antibody (Ab) were purchased from Sigma‐Aldrich Inc. (St Louis, MO, USA) and Southern Biotech (Birmingham, AL, USA), respectively, and used for Fcγ receptor stimulation. For immunoblotting (IB), we used antiphosphotyrosine (4G10) and anti‐Rac1monoclonal Abs (Merck Millipore, Billerica, MA, USA), anti‐SLP‐76, anti‐Akt, anti‐Erk2, anti‐Rap1, antiglutathione S‐transferase (GST), anti‐IκBα, and anti‐β‐actin polyclonal Abs (Santa Cruz Biotechnology Inc., Santa Cruz, CA, USA), and antiphospho‐Akt1 (p‐Akt, S473), antiphospho‐ERK1/2 (p‐ERK1/2, T202/Y204) monoclonal Abs (Cell Signaling Technology Inc., Danvers, MA, USA), and antiphospho‐SLP‐76 (p‐SLP‐76, Y145) Ab (Invitrogen, Waltham, MA, USA). The rabbit polyclonal anti‐ARAP Ab was generated as described previously.^17^ Antimouse and antirabbit horseradish peroxidase (HRP)‐conjugated IgG secondary Abs were obtained from Jackson ImmunoResearch Laboratories, Inc. (West Grove, PA, USA) and Enzo Life Sciences, Inc. (Farmingdale, NY, USA). Fluorescein isothiocyanate (FITC)‐conjugated rabbit antimouse IgG (Jackson ImmunoResearch Laboratories Inc.) and was used for immune complex (IC) formation. For the flow cytometry, FITC‐conjugated rabbit antimouse CD3, allophycocyanin (APC)‐conjugated anti‐CD11c, phycoerythrin (PE)‐conjugated anti‐CD11a, biotin‐conjugated antimouse I‐A/I‐E, and PE‐conjugated anti‐CD29 Abs from BioLegend, Inc. (San Diego, CA, USA), PE‐conjugated streptavidin from Jackson ImmunoResearch Latoratories, Inc., PE‐conjugated antimouse CD69 Ab from Invitrogen, and antimouse‐CD18 Ab from Santa Cruz Biotechnology Inc. were used. Anti‐p65 Ab from Santa Cruz Biotechnology Inc., tetramethylrhodamine isothiocyanate (TRITC)‐conjugated phalloidin from Sigma‐Aldrich Inc., and 4′,6‐diamidine‐2′‐phenylindole dihydrochloride (DAPI) (Roche Diagnostics GmbH, Mannheim, Germany) were used for the immunofluorescence. Enhanced chemiluminescence (ECL) reagent was purchased from Amersham Pharmacia Biotech Co. (Arlington Heights, IL, USA). FITC‐phalloidin, laminin, bovine serum albumin (BSA), glutathione agarose, and paraformaldehyde (PFA) were obtained from Sigma‐Aldrich Inc. In addition, human fibronectin (BD Biosciences, San Jose, CA, USA), ICAM‐1‐Fc chimera (R&D Systems Inc., Minneapolis, MN, USA), mouse IL‐6 and TNF‐α Ready‐SET‐Go!® enzyme‐linked immunosorbent assay (ELISA) sets (both from eBioscience, Inc., Santa Clara, CA, USA), mouse IL‐2 ELISA kit (Invitrogen), recombinant murine granulocyte‐macrophage colony‐stimulating factor (GM‐CSF, 315–03; Peprotech Inc., Cranbury, NJ, USA), calcein acetoxy‐methyl ester (calcein AM) and Alexa Fluor 488‐conjugated ovalbumin (AF488‐OVA) (both from Invitrogen) were used. Anti‐OVA IgG2a Ab was purchased from BioLegend, Inc. Magnetic particle‐labeled anti‐CD11cAb and CD4^+^ T‐cell isolation kit from Miltenyi Biotec (Bergisch Gladbach, Germany) were used for cell separation. Collagenase D and DNase I were obtained from Roche (Basel, Switzerland). For RNA extraction and complementary DNA (cDNA) synthesis, TRIsol® reagent (Invitrogen), oligo (dT)_15_ primer and Moloney murine leukemia virus reverse transcriptase (Promega Corporation, Madison, WI, USA) were used.

### BMDC generation

Bone marrow cells were flushed from the femora and tibiae of mice via the injection of 1 mL of sterile PBS as described previously with modifications.^37^ Cells were then resuspended in RPMI 1640 culture medium containing 10% heat‐inactivated fetal bovine serum, 50 μm 2‐mercaptoethanol, and 20 ng +mL^−1^ GM‐CSF. Fresh culture medium was added after 3 days of culture at 37°C in a 5% CO_2_ incubator. Floating cells harvested after 5 days of culture were resuspended in fresh culture medium and cultured for an additional 3 days at 37°C and 5% CO_2_. We then conducted purity analysis of the obtained BMDCs via CD11c staining and flow cytometry. More than 90% of the obtained BMDCs were used in the following experiments.

### Purification of DCs and T cells

Single‐cell suspensions were prepared from spleens by enzymatic digestion with collagenase D (2 mg mL^−1^) and DNase 1 (10 U mL^−1^) in RPMI 1640 medium at 37°C for 30 min, followed by mechanical disruption and filtration through a 70‐μm cell strainer.^38^ T‐cell suspensions were prepared from lymph nodes of OT‐II TCR transgenic mice. DCs and CD4^+^ T cells were enriched using a CD11c^+^ magnetic isolation kit (positive selection) and a CD4^+^ T‐cell isolation kit (negative selection), respectively, according to the manufacturer's instructions (Miltenyi Biotec). The purity of the isolated cell population was routinely >83% for DCs and >95% for T cells as assessed by flow cytometry.

### RNA extraction and RT‐PCR

Total RNA was extracted from the cultured BMDCs using TRIzol® reagent. We then prepared cDNA from an RNA template using oligo (dT)_15_ primers and subjected it to PCR. ARAP and GAPDH fragments were amplified, and the PCR products were separated on 3% and 1.2% agarose gels. The primer sequences were as follows: ARAP: 5′‐CAGGTGGAGCAGGTGAGGAG‐3′, 5′‐CTAGGCAGCGACTGGGAGAC‐3′; GAPDH: 5′‐ATCACCATCTTCCAGGAGGGA‐3′, 5′‐ATGACCTTGCCCACAGCCTT‐3′.

### FcγR stimulation

The BMDCs or splenic DCs were mixed on ice with mouse IgG2a (5 μg mL^−1^) for 10 min, followed by the addition of goat antimouse IgG Ab (10 μg mL^−1^) at 37°C for the durations indicated in the figures. DCs were also stimulated with RPMI 1640 medium in IgG2a‐coated plates (5 μg mL^−1^).

### Immunoblotting (IB) analysis

Immunoblotting (IB) was performed as described in a previous study.^39^ Whole‐cell lysates of BMDCs or FcγR‐stimulated BMDCs were prepared in 1% Nonidet P‐40 lysis buffer containing protease and phosphatase inhibitors. Lysates (50 μg) were mixed with 6× Laemmli sample buffer, boiled, and then subjected to 10% SDS‐PAGE. IB analysis was performed using an antimouse ARAP antibody or the indicated antibody, followed by the appropriate HRP‐conjugated secondary antibody. Chemiluminescence detection was conducted with ECL reagents.

### GTPase activation assay

The activation of Rac1 and Rap1 GTPases was assayed as previously described using the GST‐RalGDS and GST‐PAK1 plasmids.^17^, ^20^ Glutathione agarose beads were conjugated with PAK1 PBD (GST‐PBD) or RalGDS RBD (GST‐RBD). BMDCs (4 × 10^6^) were stimulated with mouse IgG2a (5 μg mL^−1^) and antimouse IgG (10 μg mL^−1^) at 37°C for the indicated durations. Cells were lysed in 500 μL of GTPase activation buffer at each time point after stimulation. Cell lysates (250 μg) were transferred to the glutathione agarose beads (GST‐PBD and GST‐RBD) for the Rac1 and Rap1 activation assays, respectively. Unstimulated conjugated beads were used as negative controls. After incubating at 4°C for 20 min, the beads were mixed with 2× Laemmli reducing buffer, boiled at 96°C for 5 min, and subjected to 14% SDS‐PAGE. Immunoblotting was performed for Rac1 or Rap1. Loading controls were observed using anti‐GST and anti‐β‐actin antibodies.

### Actin polymerization assay

BMDCs were stimulated for 24 h in plates coated with IgG2a (5 μg mL^−1^), then fixed with 4% PFA, permeabilized with 0.1% Triton X‐100, and stained with phalloidin‐FITC before measurement of the fluorescence intensity. The data were acquired at the Ewha Fluorescence Core Imaging Center using a FACSCalibur or LSR Fortessa™ cell analyzer and BD FACSDiva software v.8.0.1 (BD Biosciences).

### Immune complex uptake assay

IgG2a (1 μg mL^−1^) was mixed with FITC‐conjugated antimouse IgG Ab (3 μg mL^−1^) to form ICs. BMDCs (2 × 10^5^) or splenic DCs (8 × 10^5^) were incubated with the ICs at 4°C or 37°C for 30 min and then fixed with 4% PFA (20 min, room temperature). The fluorescence intensity analysis of the phagocytosed ICs was acquired at the Ewha Fluorescence Core Imaging Center using an LSR Fortessa™ cell analyzer and BD FACSDiva software v.8.0.1. Images of the cells were captured using a Nikon Eclipse TS2R microscope (Tokyo, Japan).

### Antigen uptake and processing assay

Alexa Fluor 488‐conjugated OVA (AF488‐OVA) was used as described previously.^40^ To generate OVA immune complexes (OVA‐ICs), AF488‐OVA and anti‐OVA IgG2a were mixed at a 1:2 (w/w) ratio and incubated at 37°C for 30 min. BMDCs (2 × 10^5^ cells) were incubated with OVA (10 μg mL^−1^) or OVA‐ICs at 37°C for 30 min, washed extensively to remove unbound antigen, and further incubated continuously in fresh medium at 37°C for the indicated times (up to 3 h) to allow intracellular trafficking and processing. Cells were then fixed with 4% PFA for 15 min at room temperature, and images were acquired using a Nikon Eclipse TS2R microscope (Tokyo, Japan).

### Antigen presentation assay

BMDCs (5 × 10^4^ cells) were pulsed with OVA or OVA‐ICs at 37°C for 3 h, washed, and cocultured with OT‐II T cells (2 × 10^5^ cells) in 96‐well round‐bottomed plates for 24 and 48 h. Cells were harvested and analyzed by flow cytometry (LSR Fortessa™, BD Biosciences). T cells were gated as CD3^+^ cells, and activation was assessed by CD69 expression, quantified as mean fluorescence intensity (MFI). Culture supernatants were collected at the indicated time points and analyzed for IL‐2 production by ELISA.

### DC‐T cell conjugation assay

BMDCs (3 × 10^5^ cells) were pulsed with OVA or OVA‐ICs at 37°C for 3 h and mixed with OT‐II T cells (6 × 10^5^ cells). Cell mixtures were applied onto coverslips and incubated at 37°C for 30 or 60 min to allow conjugate formation. Cells were fixed with 4% PFA for 20 min, permeabilized with 0.1% Triton‐X 100 for 15 min, and blocked with 3% BSA for 2 h at room temperature. Samples were then stained with TRITC‐conjugated phalloidin for 20 min to visualize actin, followed by DAPI staining prior to mounting. Fluorescence images were acquired using a Nikon Eclipse TS2R microscope (Tokyo, Japan). DC‐T cell contact areas were quantified using ImageJ by measuring the cell–cell interface (pixel^2^) or the fluorescence intensity at the interface, expressed in arbitrary units (a.u.).

### Adhesion assay

A flat‐bottomed 96‐well plate was coated with either fibronectin (5 μg mL^−1^) or ICAM‐1‐Fc (3 μg mL^−1^) and incubated at 4°C overnight. Incubation was followed by blocking using 1% BSA at room temperature for 2 h. The BMDCs or splenic DCs were incubated with calcein AM (5 μM) at 37°C for 30 min. Calcein‐labeled cells were stimulated with IgG2a (5 μg mL^−1^) and antimouse IgG (10 μg mL^−1^) at 37°C for 30 min; unstimulated cells were used as a negative control. BMDCs (1 × 10^5^) or splenic DCs (3 × 10^4^) were seeded in triplicate into the coated wells of the plate, and the plates were then incubated at 37°C for 30 min or 2 h. After washing to remove nonadherent cells, attached cells were fixed with 4% PFA (15 min, room temperature) and quantified via their fluorescence intensity using SpectraMax i3 and SoftMax® Pro Microplate Data Acquisition & Analysis Software (Molecular Devices, LLC., San Jose, CA, USA). Images of adherent cells were captured using a Nikon Eclipse TS2R microscope (Tokyo, Japan) at the Ewha Fluorescence Core Imaging Center.

### Immunofluorescence assay

BMDCs (2 × 10^5^; either untreated or treated with IgG2a and antimouse IgG Ab) were smeared onto coverslips and incubated at 37°C for 1 h. The cells were then fixed (4% PFA for 20 min), permeabilized (0.1% Triton‐X 100 for 15 min), and blocked (3% BSA for 2 h) at room temperature. Subsequently, the samples were stained with anti‐p65 antibody at 4°C overnight, followed by staining with FITC‐conjugated secondary antibody at 4°C for 2 h. DAPI staining was performed shortly before the samples were mounted onto glass slides. The fluorescence intensity was analyzed using a Nikon Eclipse TS2R microscope with NIS‐Elements BR 5.01.00 software at Ewha Fluorescence Core Imaging Center. Quantification of p65 nuclear translocation was performed based on the colocalization of p65 with DAPI staining, using consistent criteria across all conditions. Counting was performed in a blinded manner to avoid bias. A minimum of 10 cells in a microscopic field were selected blindly for analysis, and four randomly selected microscopic fields were examined per coverslip. The mean fluorescence intensity (MFI) of the nucleus/cytoplasm of each cell was recorded in addition to the immunofluorescence of each cell.

### ELISA

For the enzyme‐linked immunosorbent assay, Ready‐SET‐Go!® cytokine ELISA sets (eBioscience, Inc., Santa Clara, CA, USA) were used in‐line with the manufacturer's instructions. BMDCs (1 × 10^6^) were stimulated with 1 mL of medium for 24 h in a 24‐well, IgG2a‐coated culture plate. Culture supernatants were then added to each well of the 96‐well flat‐bottomed ELISA plate prepared for the assay, and the assay was run. Plates were then analyzed at 450 nm using a SpectraMAX 190 microplate reader (Molecular Devices, LLC.).

### Statistical analysis

One‐ or two‐way ANOVA with *post hoc* tests were employed for multiple comparisons. All statistical analyses were performed using Prism software (GraphPad Software, Inc., San Diego, CA, USA). *P* values of <0.05 were considered statistically significant. Asterisks indicate significant differences between the different groups (*P < 0.05; **P < 0.01; ***P < 0.001).

## AUTHOR CONTRIBUTIONS

**Mi Jin Yu:** Data curation; formal analysis; validation; visualization. **Jong Ran Lee:** Conceptualization; methodology; formal analysis; funding acquisition; project administration; resources; supervision; writing – original draft; writing – review and editing. **Chohee Park:** Conceptualization; investigation; writing – original draft; methodology; validation; visualization; writing – review and editing; formal analysis; data curation.

## CONFLICT OF INTEREST

The authors declare no potential conflicts of interest.

## ETHICS APPROVAL

Mice were used in accordance with a protocol approved by the Animal Protection and Use Committee of Ewha Womans University.

## Supporting information


Supplementary figure 1–11

## Data Availability

The data sets generated during and analyzed during the current study are available from the corresponding author upon reasonable request.
