# Supplementary material for: A novel adaptor protein, ARAP, orchestrates Fcγ receptor signaling in mouse dendritic cells via Rap1 activation and NF‐κB‐mediated cytokine production
Source: Immunol Cell Biol. 2026 May 24;104(7):642–55. doi: 10.1111/imcb.70133 (PMC13436211; doi:10.1111/imcb.70133)
Supplement: Supplementary file 1 [file IMCB-104-642-s001.pdf]

Supplementary figure 1

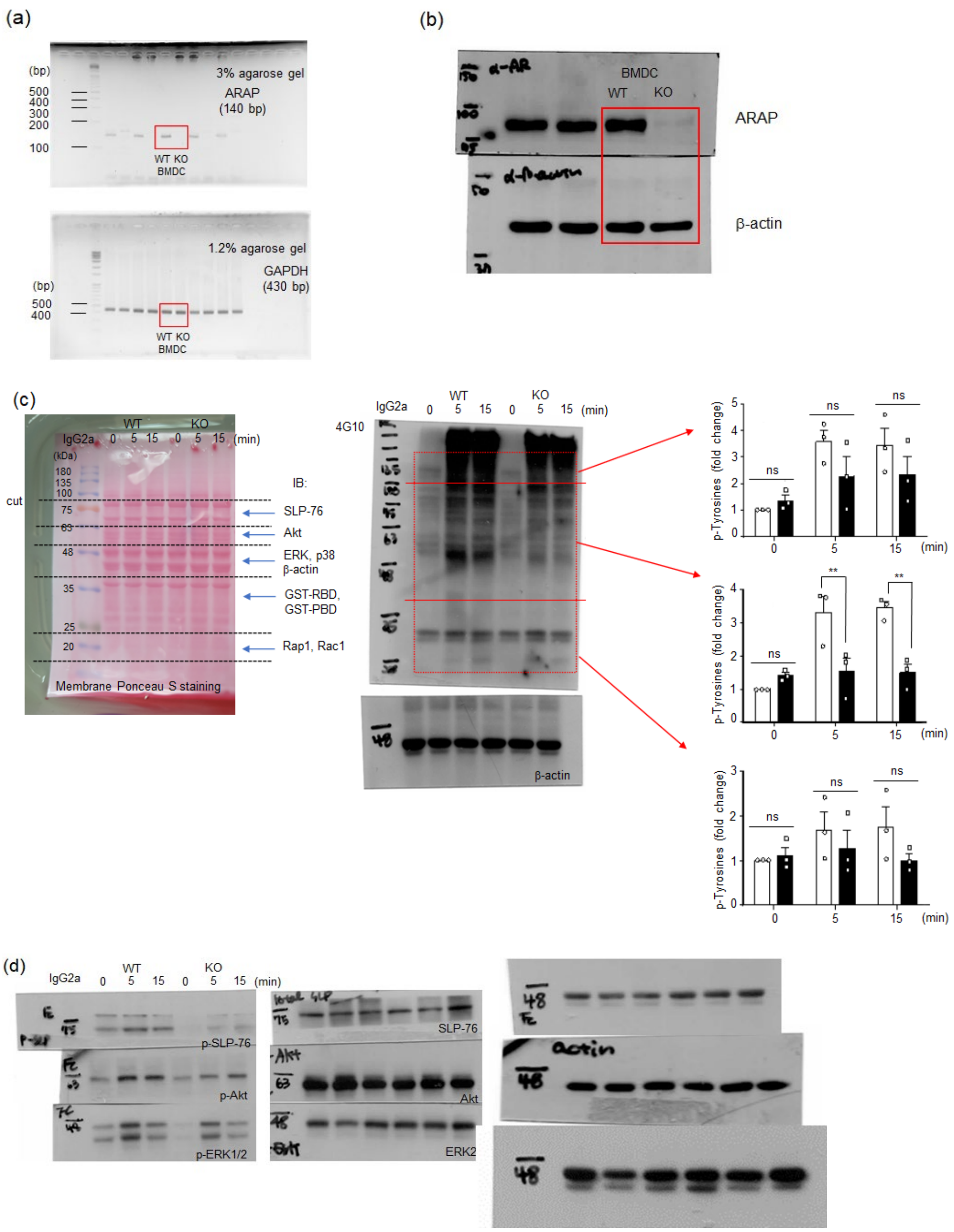

Supplementary figure 1. Original data for Figure 1. **(a, b)** The cropped regions are indicated by red rectangular boxes. **(c)** The cutting strategy of the Western blot membrane stained with Ponceau S is shown. Total tyrosine phosphorylation signals detected by 4G10 within defined molecular weight ranges (>100 kDa, 100–40 kDa, and <40 kDa) were quantitated using ImageJ. Signal intensities were normalized to β-actin as a loading control and expressed relative to unstimulated WT cells. Data are presented as mean ± SEM from three independent experiments. Statistical significance was determined by two-way ANOVA followed by Bonferroni's post-hoc tests. Significant differences are indicated as $**p < 0.01$; ns, not significant. **(d)** Original data for Figure 1d.

Supplementary figure 2

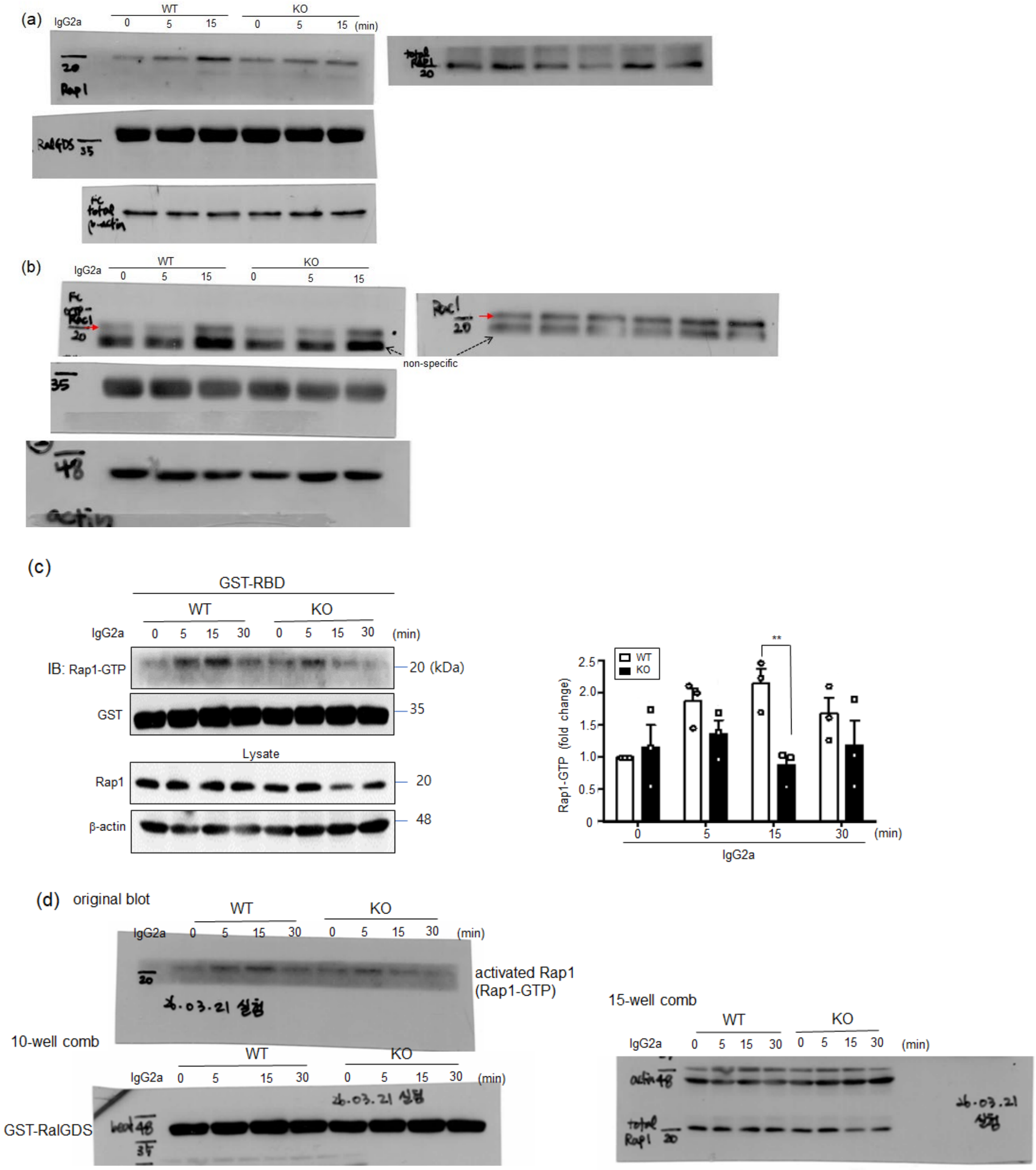

Supplementary figure 2. Original data for Figure 2 and extended time-course analysis of Rap1 activation. **(a, b)** Original data for Figure 2. **(c)** Extended time-course analysis of Rap1 activation up to 30 min. GTP-bound Rap1 (Rap1-GTP) was detected by GST-RalGDS-RBD pull-down and quantified relative to the corresponding loading control, β-actin, in whole-cell lysates. Data are presented as mean ± SEM from three independent experiments. Statistical significance was determined by two-way ANOVA followed by Bonferroni's post-hoc test. Significant differences are indicated as $^{**}p < 0.01$. **(d)** Original data for supplementary figure 2c.

Supplementary figure 3

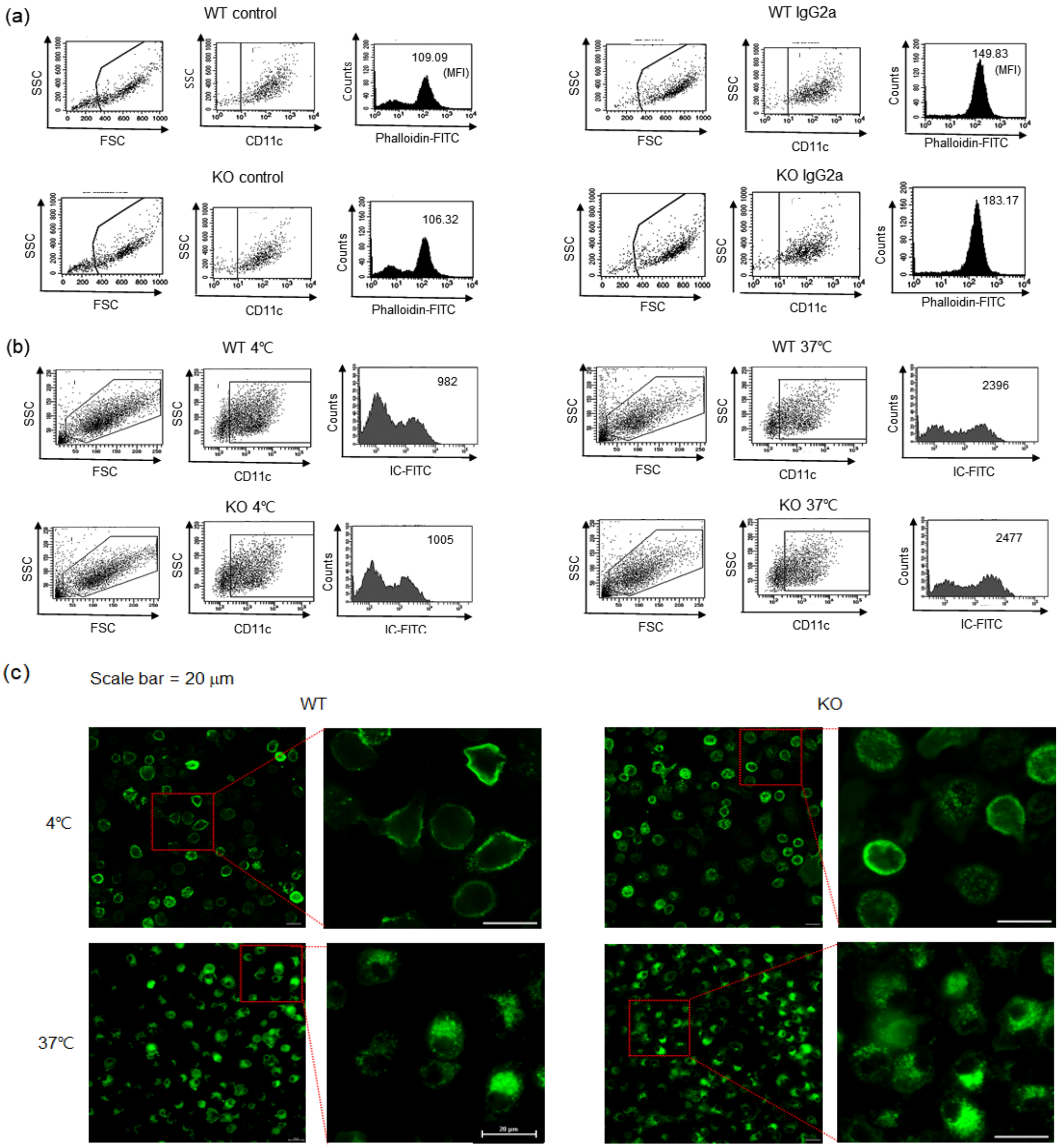

Supplementary figure 3. Original data for Figure 3. **(a, b)** Gating strategy for flow cytometry analysis. **(c)** Magnification strategy for Figure 3e.

Supplementary figure 4

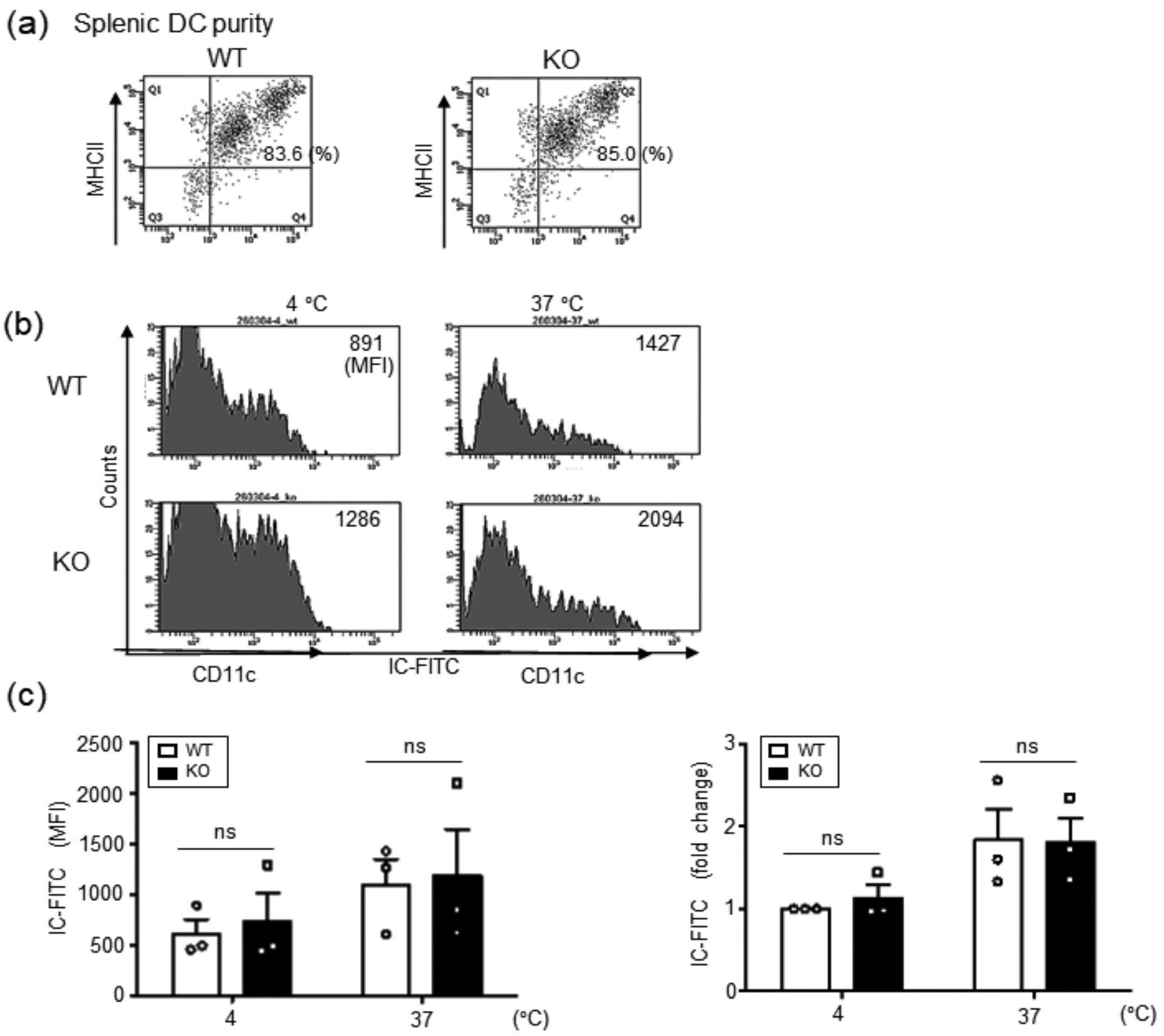

Supplementary figure 4. Immune complex (IC) uptake in DCs isolated from WT and KO spleens. **(a)** Purity of splenic DCs isolated from WT and KO mice. Flow cytometry analysis shows MHCII^{+}CD11c^{+} population. **(b)** Quantification of IC uptake by flow cytometry. **(c)** Mean fluorescence intensities (MFIs) or fold changes relative to the one of WT at 4°C are presented as mean ± SEM from three independent experiments. Statistical significance was determined by two-way ANOVA (ns, not significant).

Supplementary figure 5

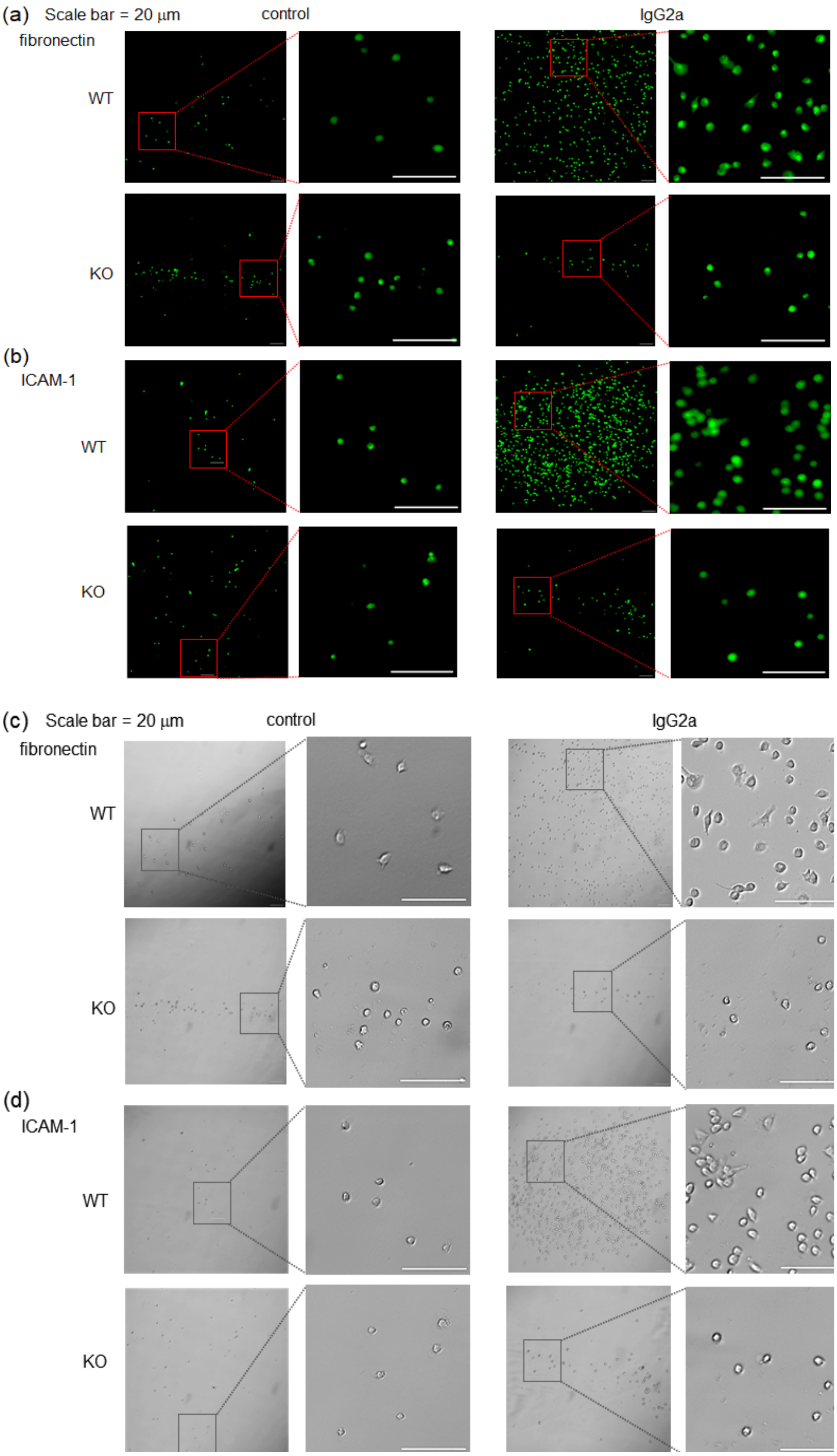

Supplementary figure 5. Original data and magnification strategy for Figure 4.

Supplementary figure 6

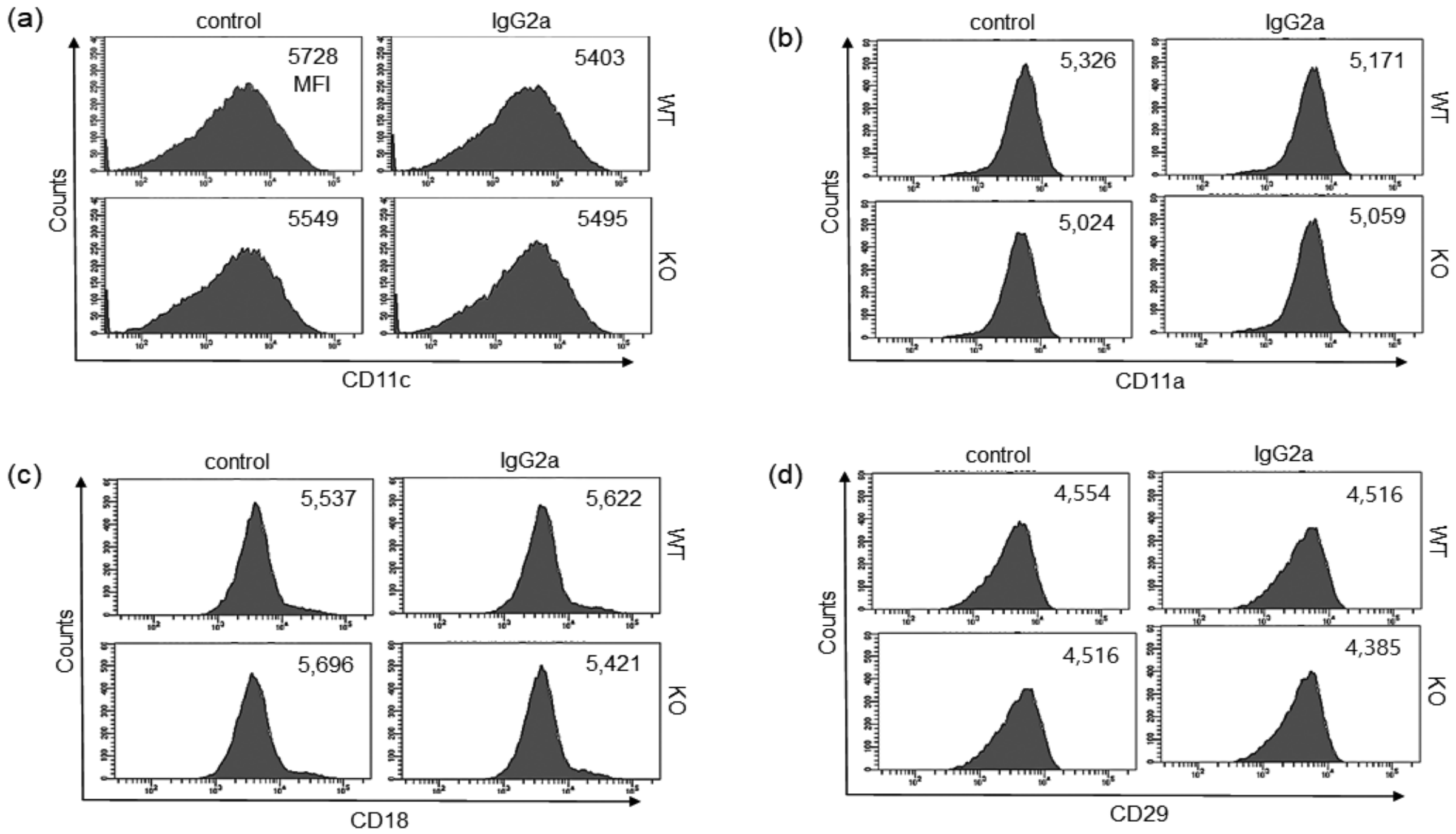

Supplementary figure 6. Expression of integrins in WT and KO BMDCs. Surface expression of major integrins and adhesion-related molecules was analyzed by flow cytometry in WT and ARAP-deficient BMDCs. Numbers indicate mean fluorescence intensity (MFI). Expression levels were comparable between WT and KO cells under both unstimulated and FcγR-stimulated conditions.

Supplementary figure 7

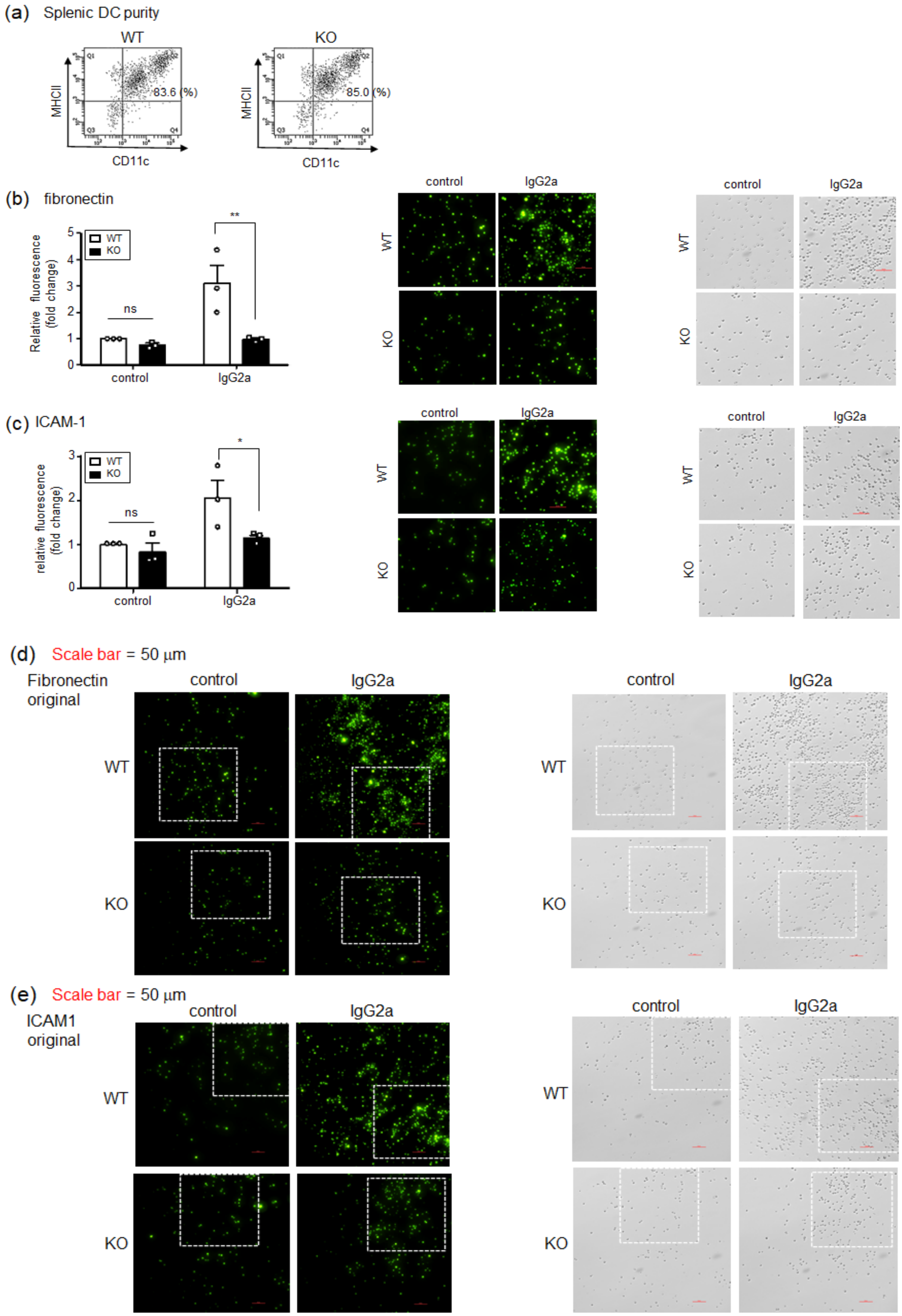

Supplementary figure 7. Adhesion defects in DCs isolated from spleens of ARAP-KO mice. **(a)** Purity of splenic DCs isolated from WT and KO mice. Flow cytometry analysis shows MHCII^{+}CD11c^{+} population. **(b, c)** Calcein-labeled WT and ARAP-KO DCs were either left unstimulated (control) or stimulated via FcγR (IgG2a) at 37°C for 30 min. Cells were then seeded onto plates coated with fibronectin **(b)** or ICAM-1-Fc **(c)**. Fluorescence intensity, which correlates with the number of adherent calcein-labeled cells, is presented as relative values normalized to unstimulated WT DCs. Each dot represents the mean of technical triplicates from a single independent experiment, and the data are compiled from three independent experiments. Data are presented as mean ± SEM. Statistical significance was determined by two-way ANOVA followed by Bonferroni's post-hoc test (*p < 0.05, **p < 0.01; ns, not significant). Adherent cells after 2 h of incubation at 37°C were further analyzed by microscopy. Representative fluorescence and corresponding bright-field images of the same samples are shown to qualitatively support the adhesion phenotype. Scale bars = 50 µm. (**d**, **e**) Original data for Supplementary figure 7b and c.

Supplementary figure 8

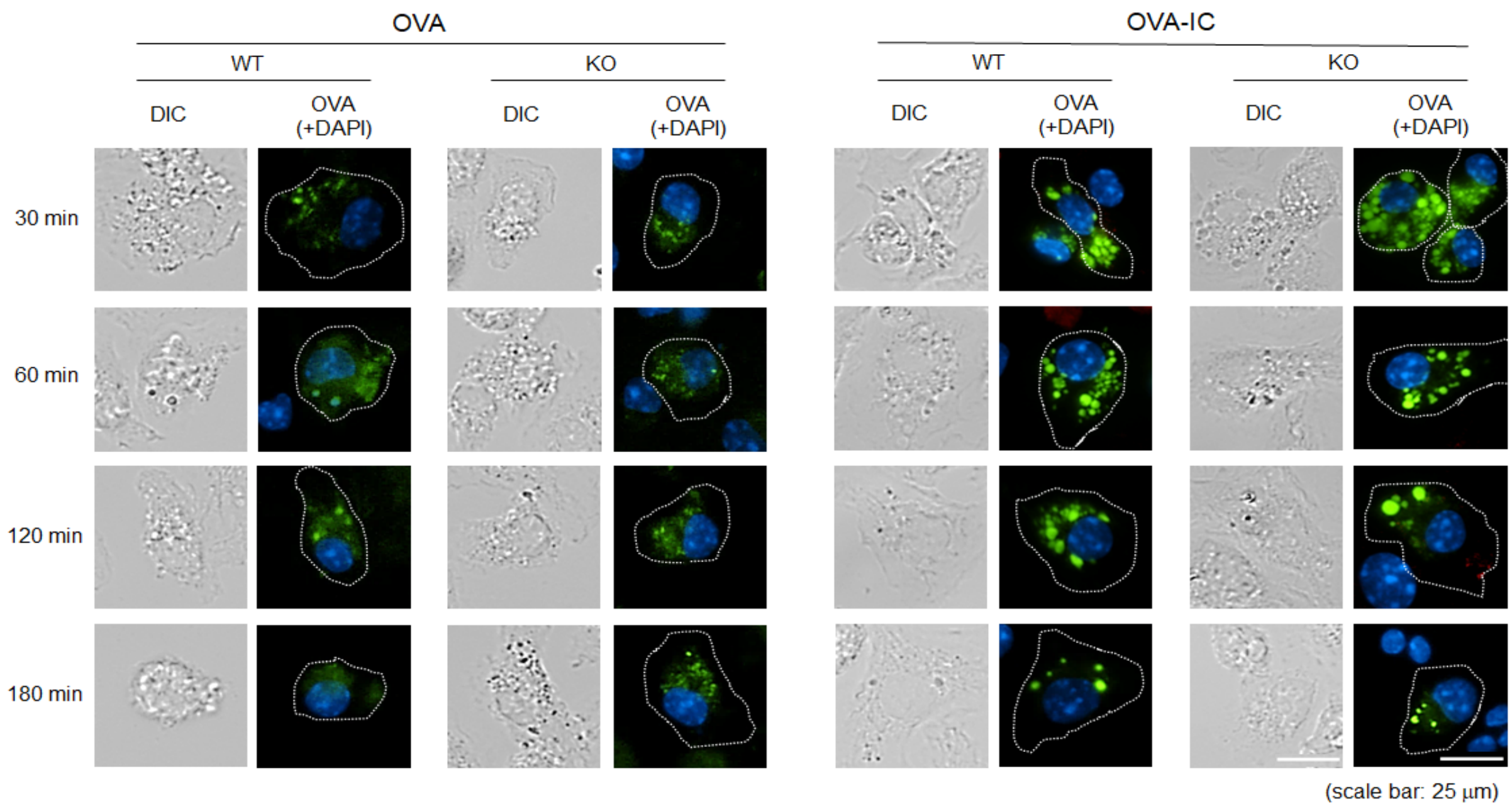

Supplementary figure 8. WT and ARAP-KO BMDCs were pulsed with AF488-OVA or AF488-OVA-ICs at 37 °C for 30 min, washed, and chased for the indicated times. Cells were analyzed at the indicated time points by fluorescence microscopy. Differential interference contrast (DIC) images of the same fields are also shown.

Supplementary figure 9

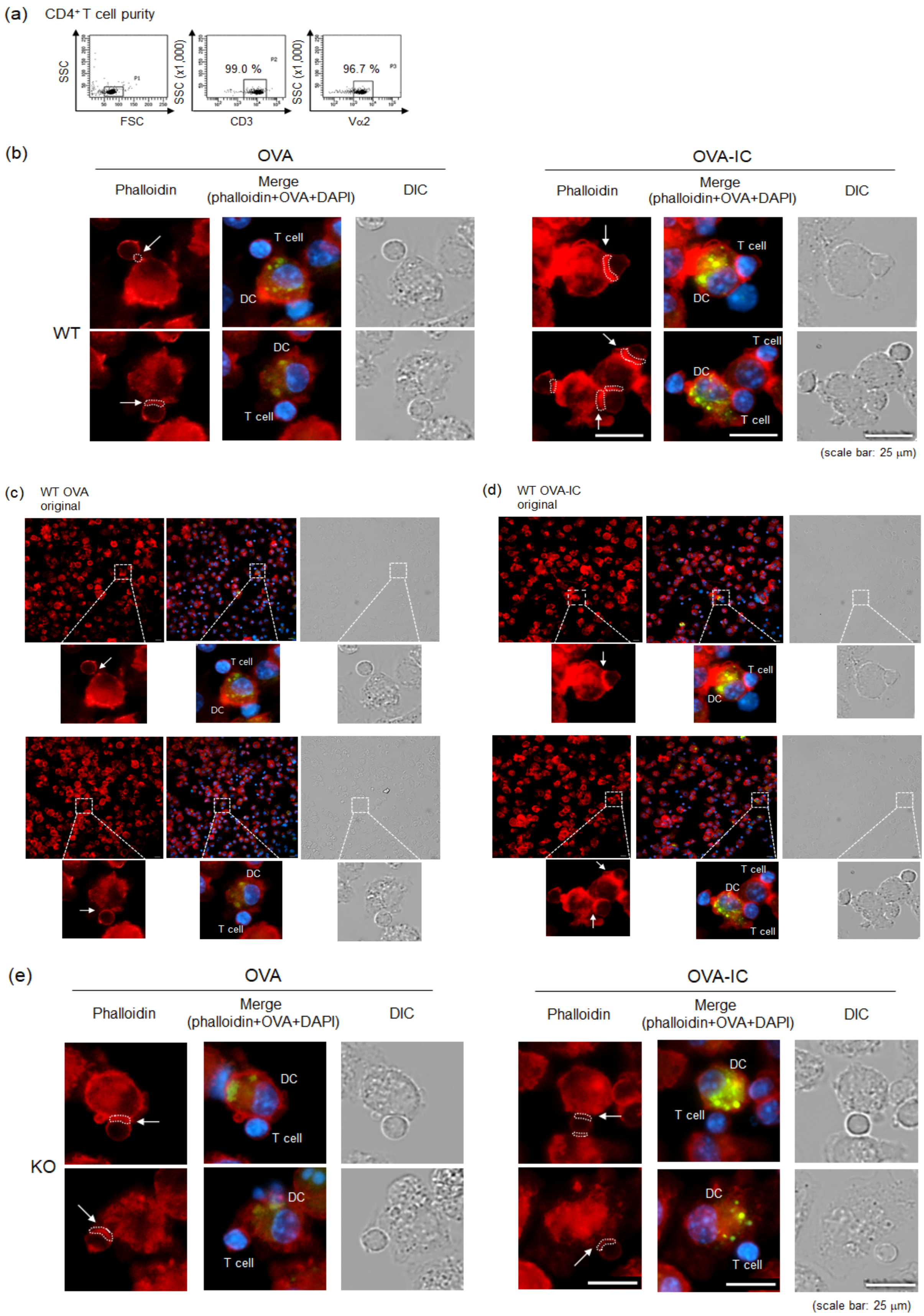

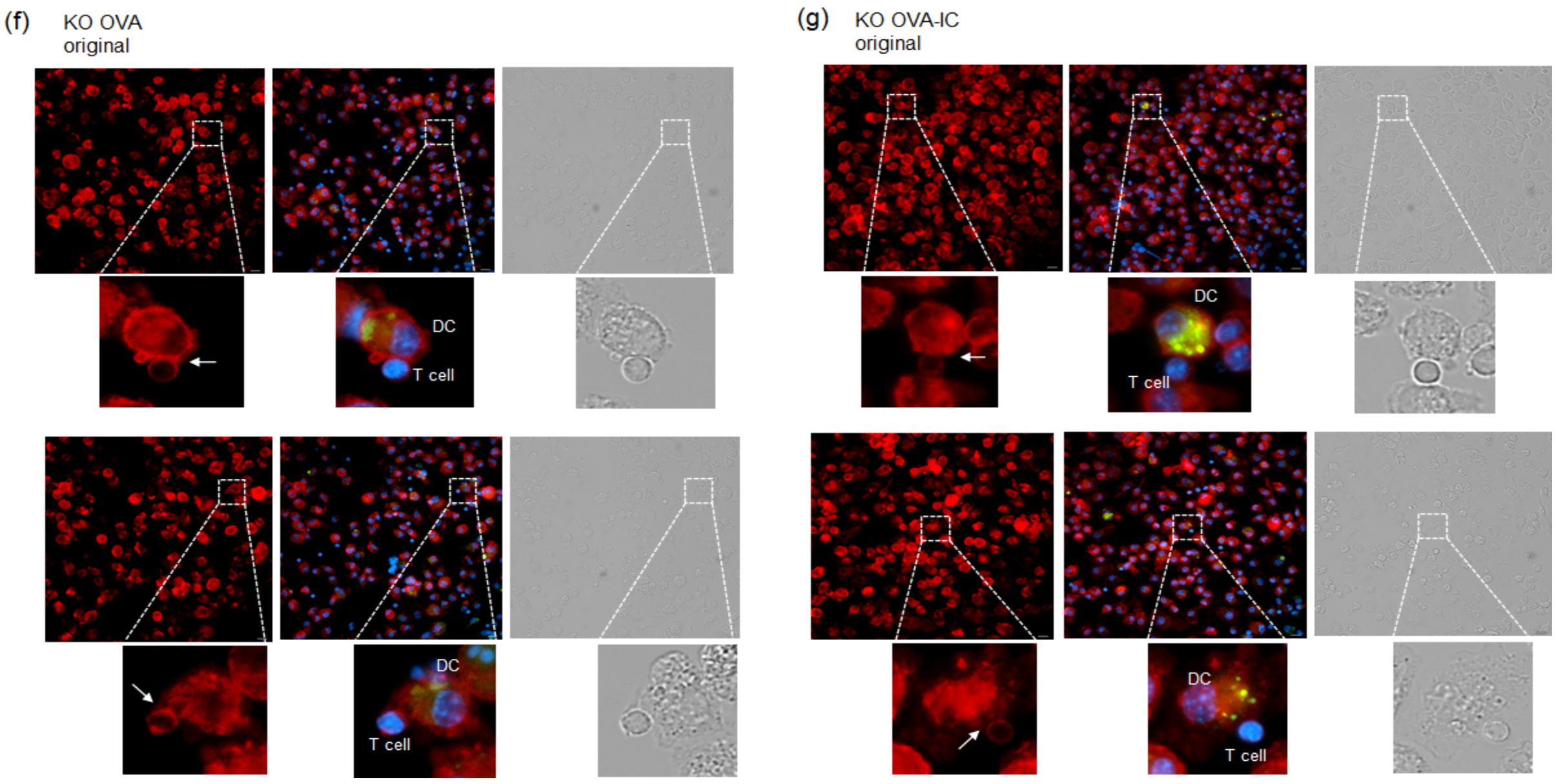

Supplementary figure 9. Analysis of DC-T cell contact. **(a)** Purity of $CD4^+$ T cells isolated from the lymph nodes of OT-II TCR transgenic mice. Flow cytometry analysis shows $V\alpha2^+$ T cell population. **(b**, **e)** WT **(b)** or KO **(e)** BMDCs pulsed with OVA or OVA-ICs were co-cultured with OT-II T cells. After 30 min, contact areas (arrows) were visualized by actin staining. Conjugated T cells were identified based on their smaller size relative to DCs. Two representative image sets show strong DC-T cell contact in WT DCs pulsed with OVA-ICs. Actin and nuclei are shown in red and blue, respectively. Scale bar = 25 μm. DC-T cell contact areas are outlined. Corresponding DIC images are also shown. **(c**, **d**, **f**, **g)** Original data and magnification strategy for DC-T cell conjugates formed with WT **(c**, **d)** or KO **(f**, **g)** DCs pulsed with OVA or OVA-IC.

Supplementary figure 10

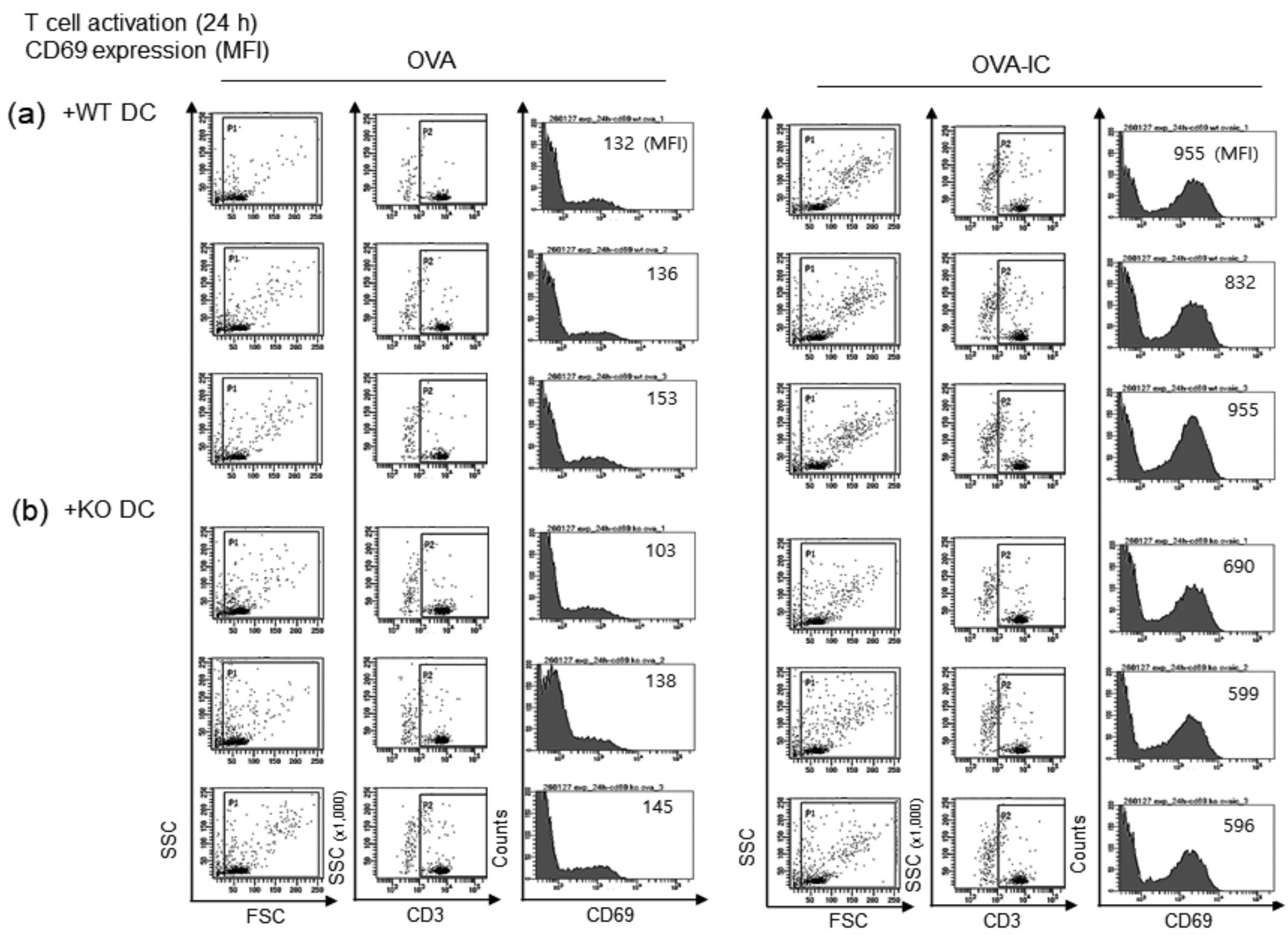

Supplementary figure 10. Flow cytometry analysis of CD69 expression in T cells co-cultured with OVA- or OVA-IC pulsed WT **(a)** or KO **(b)** BMDCs for 24 h. Numbers indicate mean fluorescence intensity (MFI).

Supplementary figure 11

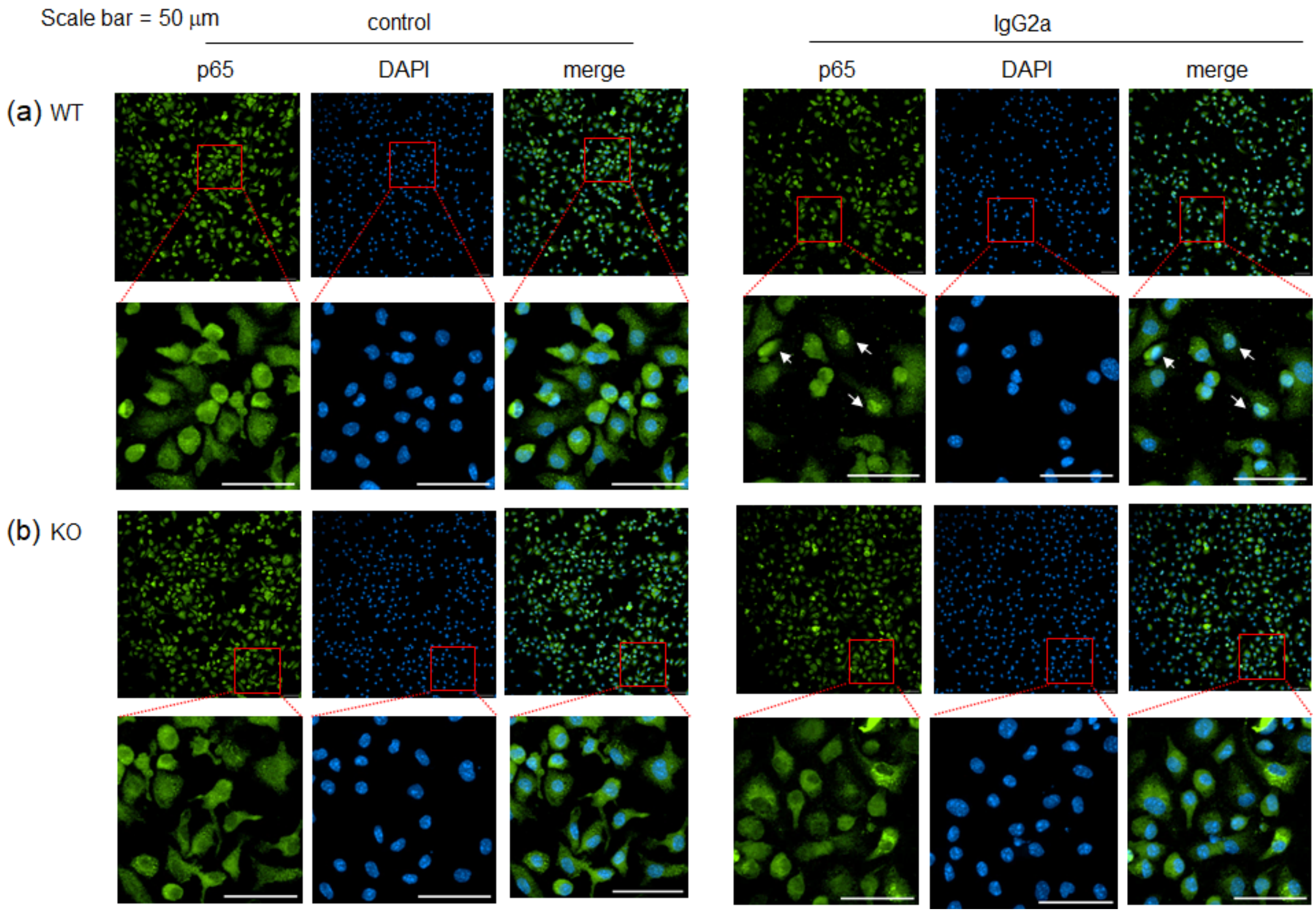

Supplementary figure 11. Original data and magnification strategy for FcγR-mediated NF-κB nuclear translocation in the BMDCs from WT **(a)** and ARAP-KO **(b)** mice. Cells were scored as p65-positive nuclei (arrows) only when the p65 signal co-localized with DAPI staining.
